# Safety of paclitaxel-coated devices in the femoropopliteal arteries: A systematic review and meta-analysis

**DOI:** 10.1371/journal.pone.0275888

**Published:** 2022-10-13

**Authors:** Chenyang Zhang, Guosheng Yin

**Affiliations:** Department of Statistics and Actuarial Science, University of Hong Kong, Hong Kong, China; Universita degli Studi Magna Graecia di Catanzaro, ITALY

## Abstract

**Background:**

Clinical benefit of paclitaxel-coated devices for patients with peripheral arterial disease has been confirmed in randomized controlled trials (RCTs). A meta-analysis published in 2018 identified late mortality risk over a long follow-up period due to use of paclitaxel-coated devices in the femoropopliteal arteries, which caused enormous controversy and debates globally. This study aims to further evaluate the safety of paclitaxel-coated devices by incorporating the most recently published data.

**Methods:**

We searched for candidate studies in PubMed (MEDLINE), Scopus, EMBASE (Ovid) online databases, government web archives and international cardiovascular conferences. Safety endpoints of interest included all-cause mortality rates at one, two and five years and the risk ratio (RR) was used as the summary measure. The primary analysis was performed using random-effects models to account for potential clinical heterogeneity.

**Findings:**

Thirty-nine RCTs including 9164 patients were identified. At one year, the random-effects model yielded a pooled RR of 1.06 (95% CI [0.87, 1.29]) indicating no difference in short-term all-cause deaths between the paclitaxel and control groups (crude mortality, 4.3%, 214/5025 versus 4.5%, 177/3965). Two-year mortality was reported in 26 RCTs with 382 deaths out of 3788 patients (10.1%) in the paclitaxel arm and 299 out of 2955 patients (10.1%) in the control arm and no association was found between increased risk of death and usage of paclitaxel-coated devices (RR 1.08, 95% CI [0.93, 1.25]). Eight RCTs recorded all-cause deaths up to five years and a pooled RR of 1.18 (95% CI [0.92, 1.51]) demonstrated no late mortality risk due to use of paclitaxel-coated devices (crude mortality, paclitaxel 18.2%, 247/1360 versus control 15.2%, 122/805).

**Conclusions:**

We found no significant difference in either short- or long-term all-cause mortalities between patients receiving paclitaxel-coated and uncoated devices. Further research on the longer-term safety of paclitaxel usage (e.g., 8- or 10-year) is warranted.

**Registration:**

PROSPERO, CRD42021246291.

## Introduction

Peripheral arterial disease (PAD) is a common cardiovascular disease which affects about 10% of the general population worldwide [1]. PAD is known as one of the leading causes of cardiovascular morbidity and mortality and the progressed PAD can result in severe impairment of functional capacity and deterioration of life quality [2] Standard percutaneous transluminal angioplasty (PTA) has been used as the first line endovascular therapy for the treatment of PAD but is associated with a high rate of vessel restenosis and limited durability in clinical efficacy [3]. In recent years, the newly developed drug-coated balloons (DCBs) and drug-eluting stents (DESs) using paclitaxel in the femoropopliteal arteries (FPAs) have shown substantial improvements in reducing restenosis, target lesion revascularization and late lumen loss [4–6].

However, the safety of long-term use of paclitaxel DCB and DES has raised great concerns. In December 2018, Katsanos et al. [7] demonstrated the association between the use of paclitaxel DCB or DES and increased long-term risk of all-cause deaths compared with PTA or bare metal stent (BMS) in their meta-analysis with 28 randomized controlled trials (RCTs). As a result, the U.S. Food and Drug Administration (FDA) issued warning letters to health care providers regarding potential risk of paclitaxel devices [8, 9] and a preliminary analysis conducted by FDA reported an approximately 50% increase in all-cause mortality for patients treated with paclitaxel-coated devices versus control [9].

The SWEDEPAD [10] and BASIL-3 [11] trials, which investigated paclitaxel-coated devices in patients with PAD, were both temporarily suspended in December 2018 due to concerns on patient safety. The BASIL-3 trial restarted patient enrollment in September 2019 according to recommendations from an independent expert advisory group [12]. An unplanned interim analysis of the SWEDEPAD trial [10] showed no difference in mortality between the paclitaxel-coated and uncoated groups at one year or during the entire follow-up period thus far, and based on these results, it was decided to resume enrollment in the SWEDEPAD trial in March 2020. Such conflicting evidence led to further controversy over risks and benefits of paclitaxel-used devices for the treatment of PAD [13].

We conducted this systematic review and meta-analysis to update findings from previous reports by including more recently published RCTs and explore short- and long-term safety issues of paclitaxel DCBs or DESs in the FPAs.

## Methods

This systematic review and meta-analysis were conducted in compliance with the Preferred Reporting Items for Systematic Reviews and Meta-Analyses (PRISMA) statement [14] and registered in the PROSPERO database (CRD42021246291; https://www.crd.york.ac.uk/prospero/).

### Study selection

We performed extensive online searches of the PubMed (MEDLINE), Scopus, EMBASE (Ovid) databases, government web archives (US Food and Drug Administration and European Medicines Agency) and oral presentations in international cardiovascular conferences for eligible studies from August 2018 to June 2022. Our analysis also included 28 RCTs investigated in Katasanos et al. [7], for which the literature search was up to August 2018. There were no restrictions on publication language or publication status. The detailed searching strategies were given in S1 Table.

We included studies which met the following inclusion criteria: (1) randomized controlled trial; (2) patients with peripheral arterial disease of the FPA; (3) head-to-head comparison between paclitaxel-coated/eluting balloons/stents and standard percutaneous transluminal angioplasty or bare metal stent; (4) follow-up period ≥1 year; (5) outcome measures of interest reported. The exclusion criteria were: (1) retrospective cohort study or non-randomized trial; (2) treatment in vessels other than FPA; (3) studies that compared paclitaxel-coated/eluting devices with other drug-coated/eluting devices.

### Data extraction and assessment of risk of bias

Assessment of eligible studies and data extraction were performed by two investigators separately and they resolved disagreements by discussion. Titles and abstracts (if available) of studies were reviewed and full texts of those meeting the inclusion criteria were further screened for eligibility. For each included trial, we collected information of the trial design, paclitaxel DCB and DES devices used in the intervention, baseline demographic characteristics and outcome measures of interest.

Two independent reviewers evaluated the quality of included RCTs using a revised Cochrane risk-of-bias tool for randomized trials (RoB 2.0) [15], which focuses on five domains of bias: bias arising from the randomization process; bias due to deviations from intended interventions; bias due to missing outcome data; bias in measurement of the outcome; and bias in selection of the reported results. Any discrepancies were resolved by discussion.

### Outcome measurement

In this meta-analysis, we focused on all-cause mortality at one year, two years and five years as safety endpoints to evaluate short- and long-term risks of paclitaxel-coated/eluting devices in the FPAs. If there were several studies (e.g., interim analysis) reporting the same trial, results extracted from the latest one were considered for quantitative analysis.

### Statistical analyses

The primary analysis investigated the number of all-cause deaths at several prespecified time points, for which the risk ratio (RR) with the corresponding 95% confidence interval (CI) was used as the summary measure. The pooled estimates were calculated by the random-effects model to account for heterogeneity due to differences in trial designs, use of paclitaxel devices and patient populations. The Mantel-Haenszel fixed-effects model and Bayesian meta-analysis under the binomial-logit framework were conducted as sensitivity analyses. The potential publication bias was visually evaluated by checking asymmetry of funnel plots [16] and statistically examined by Egger’s test [17].

Subgroup analyses were conducted to assess the influence of paclitaxel dose levels and types of paclitaxel-coated devices on patient mortality rates. We also performed a meta-regression analysis under the Bayesian binomial-logit model using the proportion of patients with chronic limb threatening ischemia (CLTI) in each arm as the covariate. Enrolled patients suffering from PAD consisted of those with CLTI and those with intermittent claudication (IC). Compared with IC, CLTI is an advanced stage of PAD with higher amputation and mortality rates [18] and might be one potential cause of heterogeneity among studies. All statistical analyses were performed using the R language version 4.0.3 (RStudio, Boston, MA) with the ‘meta’ package for frequentist meta-analysis and ‘jagsUI’ package for Bayesian meta-analysis and meta-regression.

## Results

**Fig 1:**
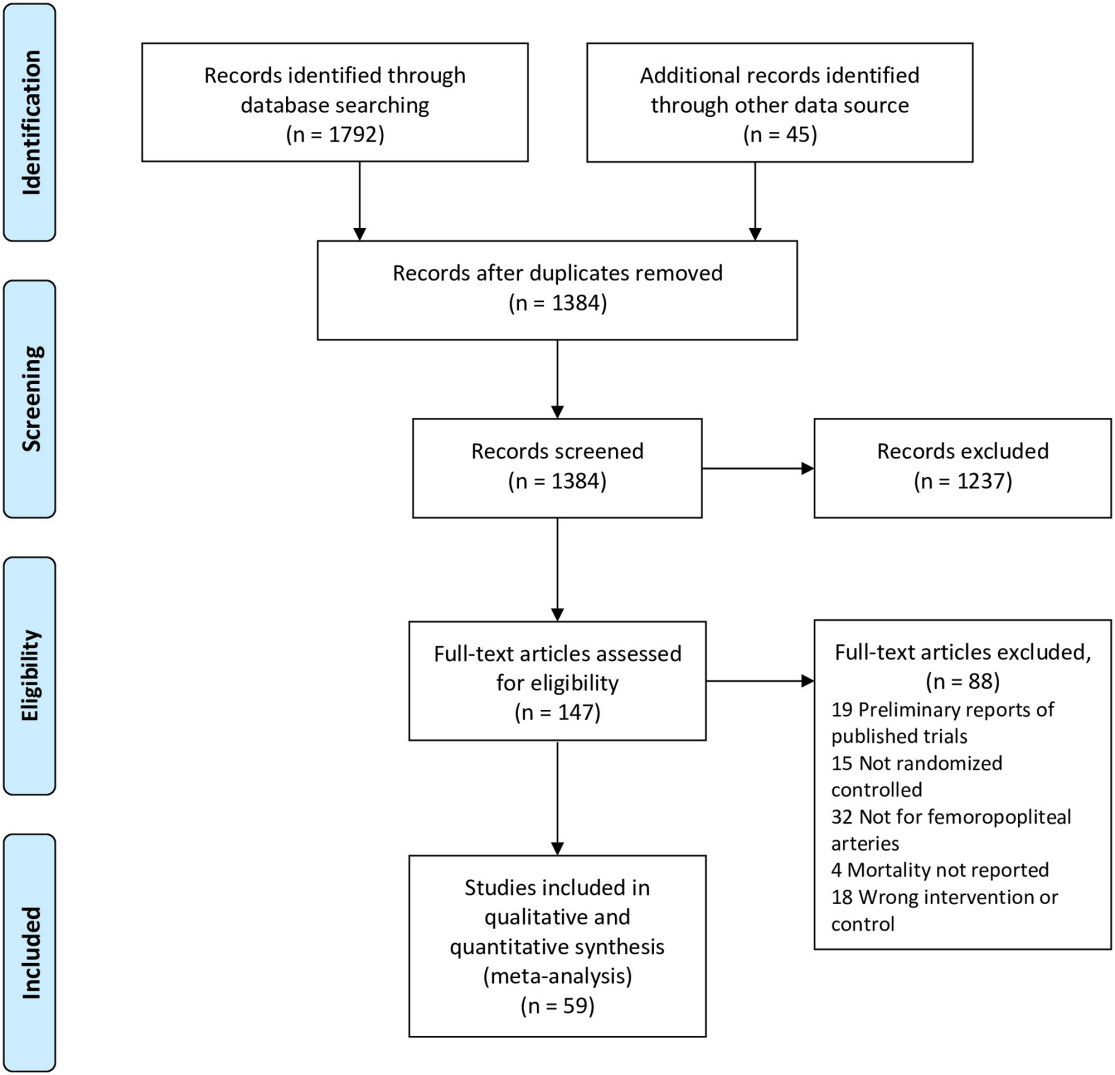
Flow diagram of study selection.

The online search from databases and other sources identified 1384 publications based on the prespecified search strategy after deleting duplicate records, of which 1237 studies were excluded after screening titles and abstracts. Full texts of the remaining 147 articles/presentations were assessed for eligibility and 59 of them were qualified for inclusion in the systematic review and meta-analysis. The flow diagram of our study selection process is shown in Fig 1.

Overall, the selected 59 studies reported 39 unique RCTs including 9164 patients [6, 10, 19–75]. The design characteristics of eligible RCTs are provided in Table 1. Out of these 39 RCTs, 29 RCTs investigated the clinical effectiveness of paclitaxel DCB, four tested the paclitaxel-coated balloon in combination with a BMS and the other six were for DES. Almost all studies evaluated the performance of one single paclitaxel device at the nominal paclitaxel dose of 2.0 (7/39), 3.0 (21/39) and 3.5 *μ*g/mm^2^ (9/39) except the SWEDEPAD trial [10] in which multiple device brands were used at various dose levels. Thirty RCTs were conducted at multiple sites, three were two-center and six were single-center. Three RCTs were double-blinded to both patients and investigators for the treatment allocation, 24 were single-blinded to patients only and the other 12 were open-label studies.

**Table 1:** Study characteristics of the 39 included randomized controlled trials in the meta-analysis.

Trial	Registration Number	Follow-up	Study Design	Location	Treatment	Control	Sample size, Intervention	Sample size, Control	Paclitaxel-Coated Device	Dose (*μ*g/mm^2^)
ZILVER-PTX [40, 59, 71]	NCT00120406	5	Multi-center; Open-label	Germany, Japan, USA	DES	PTA or BMS	241	238	ZILVER-PTX Stent by COOK Medical	3
THUNDER [24, 47]	NCT00156624	5	Multi-center; Single-blind	Germany	DCB	PTA	48	54	Cotavance Balloon by Bavaria Medizin	3
IN.PACT SFA [6, 32, 39]	NCT01175850	5	Multi-center; Single-blind	Germany	DCB	PTA	220	111	IN.PACT Admiral Balloon by Medtronic	3.5
	NCT01566461									
FEMPAC [44]	NCT00472472	2	Multi-center; Single-blind	Germany	DCB	PTA	45	42	Paccocath Balloon by Bavaria Medizin	3
LEVANT I [46]	NCT00930813	2	Multi-center; Single-blind	Belgium, Germany	DCB	PTA	49	52	Lutonix Balloon by CR BARD	2
LEVANT II [26, 72]	NCT01412541	5	Multi-center; Single-blind	Belgium, Germany, USA	DCB	PTA	316	160	Lutonix Balloon by CR BARD	2
ILLUMENATE EU [43, 49, 70]	NCT01858363	5	Multi-center; Single-blind	Austria, Germany	DCB	PTA	222	72	Stellarex Balloon by Spectranetics	2
CONSEQUENT [23, 74]	NCT01970579	2	Multi-center; Single-blind	Germany	DCB	PTA	78	75	SeQuent Please Balloon By B.Braun Melsungen AG	3
ISAR-STATH [62]	NCT00986752	2	Two-center; Open-label	Germany	DCB+BMS	PTA+BMS	48	52	IN.PACT Admiral Balloon by Medtronic	3.5
ISAR-PEBIS [45]	NCT01083394	2	Two-center; Open-label	Germany	DCB	PTA	36	34	IN.PACT Admiral Balloon by Medtronic	3.5
IN.PACT SFA JAPAN [33, 34]	NCT01947478	2	Multi-center; Single-blind	Japan	DCB	PTA	68	32	IN.PACT Admiral Balloon by Medtronic	3.5
ACOART I [22, 29, 48]	NCT01850056	5	Multi-center; Single-blind	China	DCB	PTA	100	100	Orchid Balloon by Acotec Scientific	3
FINN-PTX [37]	NCT01450722	2	Multi-center; Open-label	Finland	DES	PTFE Bypass Graft	23	18	ZILVER-PTX Stent by COOK Medical	3
BATTLE [61]	NCT02004951	2	Multi-center; Open-label	France	DES	BMS	86	85	ZILVER-PTX Stent by COOK Medical	3
DEBATE-IN-SFA [38]	UMIN000010071	1	Multi-center; Open-label	Japan	DES	BMS	85	85	ZILVER-PTX Stent by COOK Medical	3
DEBELLUM [27]	NA	1	Single-center; Open-label	Italy	DCB	PTA	25	25	IN.PACT Admiral Balloon by Medtronic	3.5
PACIFIER [57, 75]	NCT01083030	2	Multi-center; Single-blind	Germany	DCB	PTA	44	47	IN.PACT Pacific Balloon by Medtronic	3.5
FAIR [31]	NCT01305070	1	Multi-center; Single-blind	Germany	DCB	PTA	62	57	IN.PACT Admiral Balloon by Medtronic	3.5
BIOLUX P-I [60]	NCT01056120	1	Multi-center; Single-blind	Austria,Belgium,France,Germany,Ireland,Israel,Latvia,Netherlands,Spain,Switzerland	DCB	PTA	30	30	Passeo-18 Lux Balloon by Biotronik	3
RANGER SFA [21, 69]	NCT02013193	1	Multi-center; Single-blind	Austria,France,Germany	DCB	PTA	71	34	Ranger Balloon by Boston Scientific	2
ILLUMENATE pivotal [43, 54, 68]	NCT01858428 NCT01912937	5	Multi-center; Single-blind	USA	DCB	PTA	200	100	Stellarex Balloon by Spectranetics	2
DEBATE-SFA [36]	NCT01556542	1	Single-center; Open-label	Italy	DCB+BMS	PTA+BMS	53	51	IN.PACT Admiral Balloon by Medtronic	3.5
LEVANT JAPAN [50, 66]	NCT01816412	2	Multi-center; Single-blind	Japan	DCB	PTA	71	38	Lutonix Balloon by CR BARD	2
RAPID [28, 67]	ISRCTN47846578	2	Multi-center; Double-blind	Netherlands	DCB+BMS	PTA+BMS	80	80	Legflow Balloon by Cardionovum	3
EFFPAC [30, 41, 53]	NCT02540018	2	Multi-center; Single-blind	Germany	DCB	PTA	85	86	Luminor-35 Balloon by iVascular	3
PACUBA [58]	NCT01247402	1	Two-center; Single-blind	Austria	DCB	PTA	35	39	FREEWAY Balloon by Eurocor	3
FREEWAY [64]	NCT01960647	1	Multi-center; Single-blind	Austria,Germany	DCB+BMS	PTA+BMS	105	99	FREEWAY Balloon by Eurocor	3
DRECOREST [35]	NCT03023098	1	Single-center; Double-blind	Finland	DCB	PTA	29	28	IN.PACT Balloon by Medtronic	3.5
SWEDEPAD [10]	NCT02051088	2.49 (Mean, Ongoing)	Multi-center; Open-label	Sweden	DCB	PTA	1149	1140	Multiple device bands	NA
Falkowski et al. [25]	NA	3	Single-center; NA	Poland	DES	BMS	126	130	Zilver PTX Stent by Cook Medical	3
COPA CABANA [56]	NCT01594684	2	Multi-center; Double-blind	Germany	DCB	PTA	47	41	Cotavance Balloon by MEDRAD	3
Liao et al. [63]	ChiCTR1800017055	1	Single-center; Single-blind	China	DCB	PTA	38	36	Orchid Balloon by Acotec Scientific	3
RANGER II SFA [19, 20]	NCT03064126	1	Multi-center; Single-blind	Austria,Belgium,Canada,Japan,New Zealand,USA	DCB	PTA	278	98	Ranger Balloon by Boston Scientific	2
BIOPAC [51]	NCT02145065	3	Multi-center; Single-blind	Poland	DCB	PTA	33	33	Microcrystalline PAK Balloon by Balton Sp. z o.o., Warszawa, Poland	3
Ni et al. [52]	NCT03844724	1	Multi-center; Single-blind	China	DCB	PTA	93	99	ZENFlow Balloon by Zylox Medical Device Inc	3
ORCHID CHINA [55]	ChiCTR1900023619	1	Single-center; Single-blind	China	DCB	PTA	30	30	Orchid Balloon by Acotec Scientific	3
Ye et al. [65]	NA	2	Multi-center; Open-label	China	DCB	PTA	100	100	Reewarm™ PTX by Endovastec Co., Ltd	3
FREEWAY-CHINA [73]	NA	1	Multi-center; Open-label	China	DCB	PTA	155	154	FREEWAY Balloon by Eurocor	3
EMINENT [42]	NCT02921230	1	Multi-center; Single-blind	Austria,Belgium,France,Germany,Ireland,Italy,Netherlands,Spain,Switzerland,UK	DES	BMS	508	267	ELUVIA Stent by Boston Scientific	0.167

BMS: bare metal stent; DCB: drug-coated balloon; DES: drug-eluting stent; PTA: percutaneous transluminal angioplasty

There were three 3-arm RCTs (DEBATE-IN-SFA [38], ISAR-STATH [62] and THUNDER [24, 47]) and only patients receiving the paclitaxel DCB/DES and standard PTA/BMS were included in the meta-analysis, while observations from the BMS plus cilostazol (DEBATE-IN-SFA), directional atherectomy (ISAR-STATH) and PTA plus paclitaxel in the contrast medium (THUNDER) groups were removed. In the ZILVER-PTX trial, patients assigned to the PTA arm through the primary randomization underwent a secondary randomization to DES or BMS if PTA failed acutely [40, 59, 71] and we pooled the results of patients receiving DES during both randomizations in the analysis.

The average age was over 65 years in all studies and about 65% of patients were male. The number of patients with IC and CLTI at baseline based on the Rutherford classification was reported in 37 RCTs. In total, 2342 (29%) patients had CLTI and 5729 (71%) had IC. The majority of patients in the DEBATE-SFA [36], SWEDEPAD [10] and Ni et al. [52] trials had CLTI, and in 28 RCTs, IC only accounted for less than 20% of enrolled patients. The detailed demographic and angiographic characteristics at baseline of the included RCTs can be found in S2 and S3 Tables, respectively. All-cause mortality data at one, two and five years are shown in S4 Table.

All the included 39 RCTs were judged to be of high overall risk of bias and the main source of risk arose from the bias due to deviations from intended interventions. There existed noticeable visual difference between paclitaxel-coated/eluting devices and standard uncoated devices. Therefore, investigators were usually not blinded to treatment assignment, which might affect the clinical decision making during the follow-up period. Detailed risk of bias assessment for each included RCT is presented in S1 Fig.

### All-cause mortality

**Fig 2:**
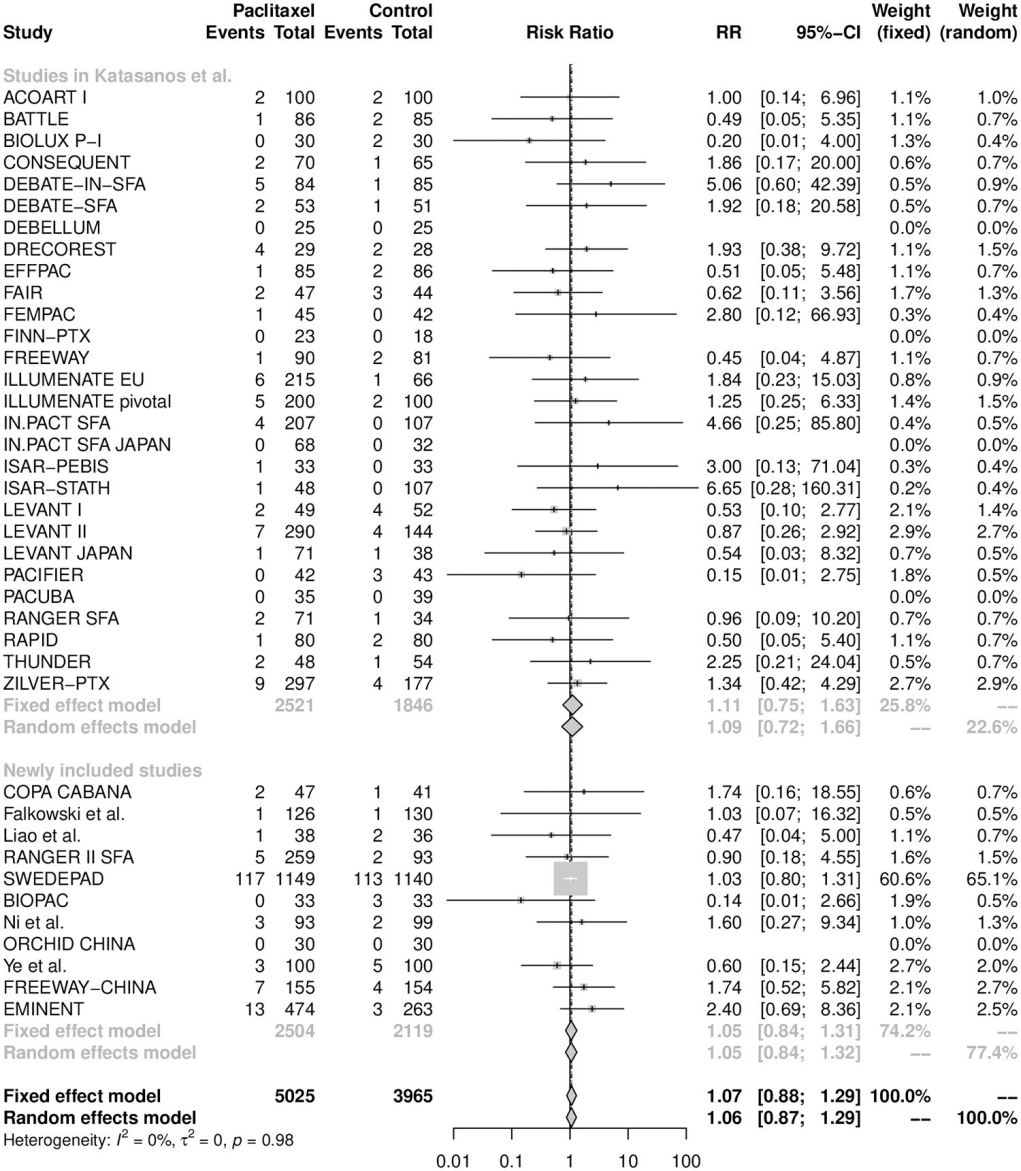
Forest plot of all-cause mortality at one year. RR is the risk ratio and CI represents the 95% confidence interval.

The one-year all-cause mortality was reported in all 39 RCTs with 8990 patients. The crude risk of death at one year was 4.3% (214/5025) in the paclitaxel arm and 4.5% (177/3965) in the control arm. As shown in Fig 2, the random-effects model yielded a pooled RR of 1.06 (95% CI [0.87, 1.29]), suggesting no statistically significant difference in one-year mortality between the paclitaxel DCB/DES and control groups. There was no heterogeneity across the studies (I^2^ = 0%; p = 0.98).

**Fig 3:**
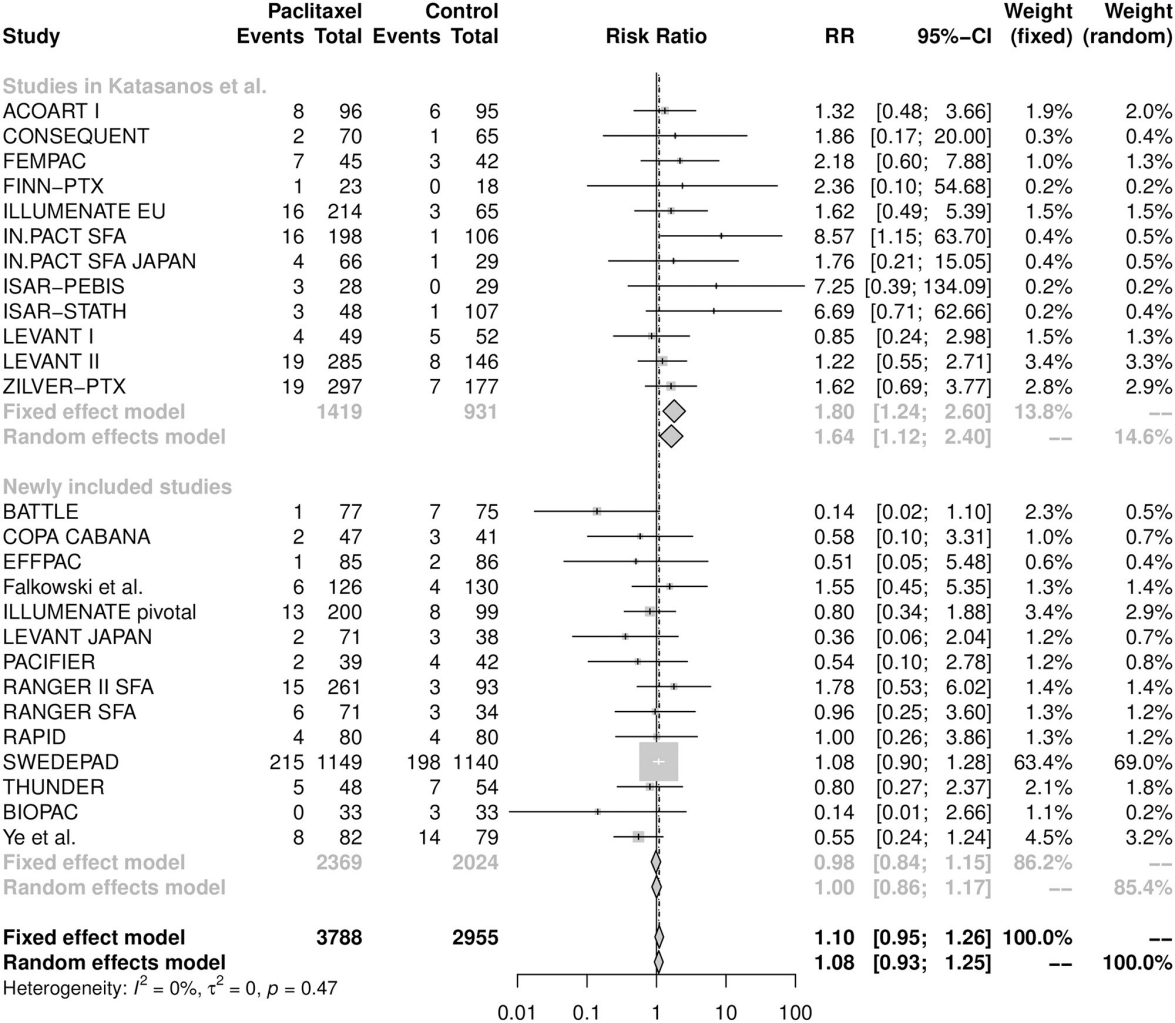
Forest plot of all-cause mortality at two years. RR is the risk ratio and CI represents the 95% confidence interval.

A total of 26 RCTs directly recorded all-cause deaths by two years. The unplanned interim analysis of SWEDEPAD trial [10] reported safety outcomes of paclitaxel-coated devices during a mean follow-up of 2.5 years. We estimated the number of deaths by two years in the SWEDEPAD trial [10] using the two-year cumulative incidence rate multiplied with the total number of patients in each group. Overall, at the two-year follow-up, 382 out of 3788 patients (10.1%) in the paclitaxel arm and 299 out of 2955 patients (10.1%) in the control arm had died. The forest plot in Fig 3 indicates that the use of paclitaxel-coated/eluting devices in the FPAs tended to increase the risk of death at two years but the evidence was not statistically significant (the random-effects model yielded RR = 1.08, 95% CI [0.93, 1.25]). We observed no heterogeneity among these 26 RCTs (I^2^ = 0%; p = 0.47).

**Fig 4:**
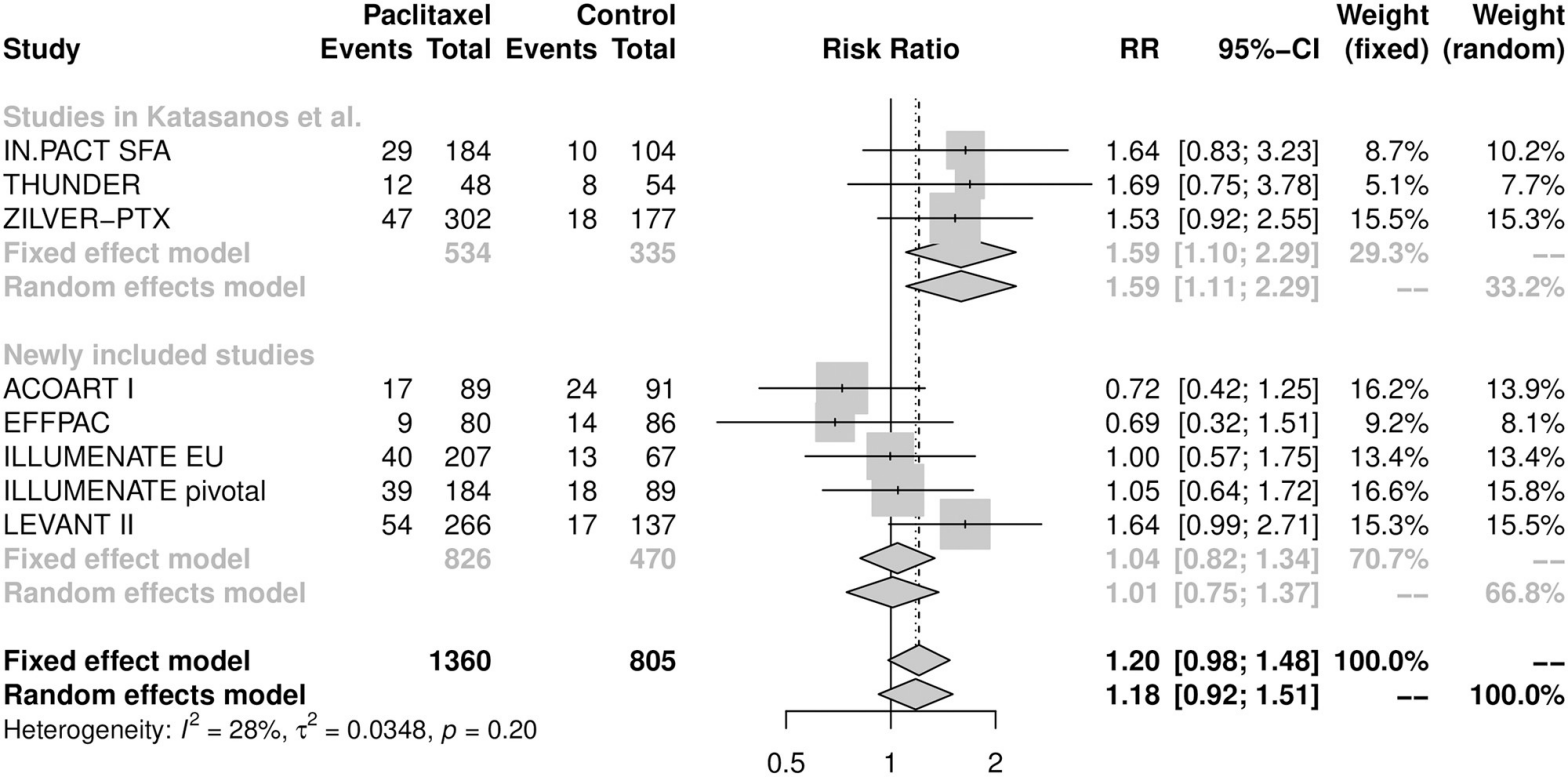
Forest plot of all-cause mortality at five years. RR is the risk ratio and CI represents the 95% confidence interval.

The five-year all-cause mortality was available only from eight RCTs with 2165 patients. The crude all-cause mortality at five years was 18.2% (247/1360) for patients receiving paclitaxel-coated/eluting devices and 15.2% (122/805) for those in the control group. The pooled RR calculated from the random-effects model was 1.18 (95% CI [0.92, 1.51]) (Fig 4). Although there was a 3.0% difference in the crude five-year mortality rate (95% CI [-0.2%, 6.2%]) between the paclitaxel-coated/eluting devices and control groups, the meta-analysis suggested that use of paclitaxel-coated balloons and stents did not significantly increase the risk of death during the five-year follow-up period. No heterogeneity was found among these eight RCTs (I^2^ = 28%; p = 0.20).

### Subgroup and sensitivity analysis

Subgroup analyses were performed to verify the influence of different paclitaxel interventions (DCB, DES or DCB+BMS) and dose levels (2.0, 3.0, 3.5*μ*g/mm^2^ stents or balloons) on all-cause mortality. We only analyzed subgroups with more than three RCTs. In subgroup analysis concerning different types of paclitaxel interventions, patients treated with DES had relatively higher one-year mortality rates compared with the control and in all intervention subgroups there was no significant difference in the risk of deaths at one, two and five years between the paclitaxel and control arms (Table 2). With respect to subgroup analysis of paclitaxel doses, studies using 3.5*μ*g/mm^2^ DCB showed a higher RR (the random-effects model yielded RR = 2.77, 95% CI [0.84, 9.22]) of two-year all-cause deaths compared to those using lower dose balloons and stents, while for all dose levels, no statistically significant result was found under the random-effects model.

**Table 2:** Subgroup analyses of all-cause mortality with subgroups including more than three randomized controlled trials.

		Risk Ratio (95% CI)	
	Period	Random-effects	Fixed-effects
Paclitaxel intervention			
DCB	1 year	1.02 (0.83, 1.26)	1.01 (0.82, 1.24)
DCB	2 years	1.06 (0.91, 1.23)	1.08 (0.93, 1.26)
DCB	5 years	1.12 (0.86, 1.48)	1.15 (0.92, 1.43)
DES	1 years	1.71 (0.83, 3.51)	1.79 (0.89, 3.59)
DES	2 years	1.09 (0.41, 2.94)	1.11 (0.61, 2.01)
DCB+BMS	1 year	1.07 (0.30, 3.76)	1.10 (0.36, 3.35)
Dose level			
2.0*μ*g/mm^2^ DCB	1 year	0.90 (0.47, 1.74)	0.92 (0.48, 1.75)
2.0*μ*g/mm^2^ DCB	2 years	1.05 (0.69, 1.58)	1.07 (0.71, 1.61)
3.0*μ*g/mm^2^ DES	1 year	1.43 (0.59, 3.48)	1.53 (0.66, 3.54)
3.0*μ*g/mm^2^ DES	2 years	1.09 (0.41, 2.94)	1.11 (0.61, 2.01)
3.0*μ*g/mm^2^ DCB	1 year	0.94 (0.55, 1.63)	0.89 (0.54, 1.48)
3.0*μ*g/mm^2^ DCB	2 years	0.86 (0.56, 1.33)	0.85 (0.56, 1.29)
3.5*μ*g/mm^2^ DCB	1 year	1.42 (0.60, 3.40)	1.35 (0.63, 2.90)
3.5*μ*g/mm^2^ DCB	2 years	2.77 (0.84, 9.22)	3.05 (1.33, 7.01)

BMS: bare metal stent; CI: confidence interval; DCB: drug-coated balloon; DES: drug-eluting stent.

We conducted sensitivity analyses using the fixed-effects model, various continuity correction methods for rare events and the Bayesian binomial-logit model on arm-level observations. We also considered exclusion of SWEDEPAD trial in the two-year meta-analysis because the data were estimated from the survival curves. As shown in S5 Table, in all cases the pooled estimate was close to that in the primary analysis and there was still no statistically significant increase of all-cause mortality due to the use of paclitaxel.

**Fig 5:**
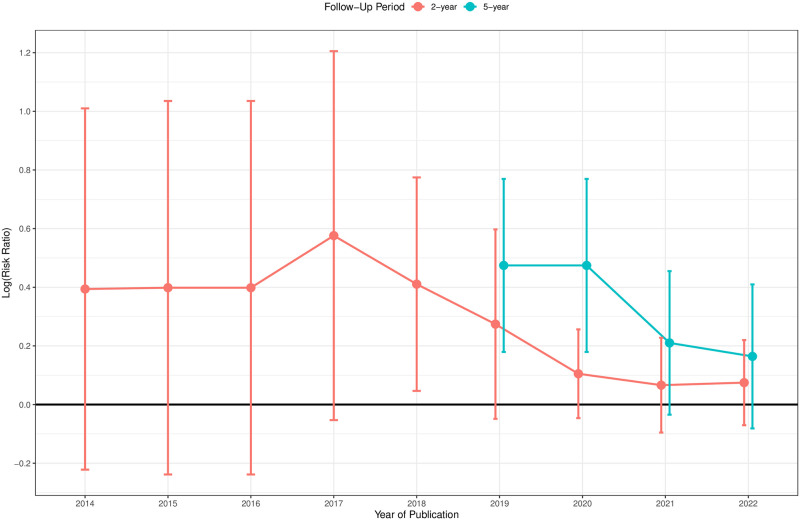
Cumulative pooled treatment effect estimates (the logarithm of risk ratio) from the random-effects model by years of publications.

In addition, we conducted cumulative meta-analysis by years of publications on the two- and five-year all-cause mortality as a post-hoc analysis and the estimates from the random-effects model were shown in Fig 5 and S6 Table. For each follow-up period, the sequential plot started from the year in which ≥3 RCTs reported the endpoint of interest. As shown in Fig 3, for the 2-year all-cause mortality, studies in Katasanos et al. [7] reported an RR of 1.64 (95% CI [1.12, 2.40]) while the newly added studies yielded an RR of 1.00 (95% CI [0.86; 1.17]), which delivered contradictory results. The difference in the two-year all-cause mortality was statistically significant only in 2018 (RR = 1.51, 95% CI [1.05, 2.17] from 12 RCTs), and the two-year RR had been falling since 2017. Four RCTs (IN.PACT SFA [6], LEVANT II [26], THUNDER [24] and ZILVER-PTX [40]) reported five-year all-cause deaths by 2019, which yielded a pooled RR of 1.61 (95% CI [1.20, 2.16]). However, the pooled results turned to be insignificant after inclusion of four newly published RCTs (ACOART I [48], ILLUMENATE EU, ILLUMENATE pivotal [43] and EFFPAC [53]) in 2021 and 2022.

### Publication bias

Visual inspection of funnel plots (S2 Fig) suggested no publication bias at one, two and five years due to approximately symmetrical shapes, and Egger’s test produced confirmative results (one-year, p = 0.87; two-year, p = 0.82; five-year, p = 0.89), see S7 Table.

## Discussion

This systematic review and meta-analysis evaluated safety of the use of paclitaxel-coated/eluting devices in the FPAs and showed no significant difference in either short- or long-term all-cause mortality between patients receiving paclitaxel DCBs/DESs and those receiving the control (one-year RR, 1.06, 95% CI [0.87, 1.29]; two-year RR 1.08, 95% CI [0.93, 1.25]; five-year RR 1.18, 95% CI [0.92, 1.51]). Our findings are contrary to the results in Katsanos et al. [7] published in 2018, which reported significant mortality signals at two and five years due to paclitaxel usage. Compared with previous meta-analyses, our study included more recently published RCTs after 2020 and we can further investigate the trend of the increased risk of death due to paclitaxel over the publication time. The cumulative meta-analysis by years of publications (Fig 5) demonstrated that the inclusion of the most recently published studies reduced the difference in mortality between the paclitaxel and control arms and thus led to insignificant pooled results. For several recent studies, e.g., BATTLE (2-year) [61], COPA CABANA (2-year) [56], EFFPAC (2-year, 5-year) [30, 53], ACOART I (5-year) [48], patients in the control arm even had a higher risk of death compared with those receiving paclitaxel interventions. Pooled estimates from the meta-analysis are consistent across various statistical methods in the sensitivity analysis. The subgroup of the high paclitaxel dose (3.5*μ*g/mm^2^) showed a potential tendency towards a higher risk of death at one and two years compared with the low-dose one. No publication bias existed at any of the investigated follow-up periods.

The controversial meta-analysis of Katsanos et al. [7] has provoked heated discussions and debates worldwide on the long-term safety of paclitaxel-delivery devices in treating PAD. Subsequently, multiple studies were performed based on different data sources and statistical models, which repeatedly identified the late mortality signal. As a result, the US FDA released three warning letters and also conducted a preliminary analysis for the FDA-approved paclitaxel-coated devices, which confirmed the finding of an increased mortality rate due to the use of paclitaxel up to five years (RR = 1.72, 95% CI [1.25, 2.38]), but no significant difference was found in the two-year mortality (RR = 1.31, 95% CI [0.76, 2.29]) [8, 9, 26]. A Bayesian meta-analysis [76] identified only a borderline difference in mortality between paclitaxel and control arms beyond two years with inconclusive evidence. To overcome the lack of access to the original patient-level data, Albrecht et al. [75] and the Vascular Interventional Advances (VIVA) group [77] performed individual-patient-data meta-analyses based on patient-level data of four and eight RCTs, respectively. Albrecht et al. [75] indicated no evidence of increased risk of death using paclitaxel interventions at two years. The VIVA group reported a hazard ratio of 1.38 (95% CI [1.06, 1.80]) during a median follow-up of four years [77]. Klumb et al. [5] conducted a meta-analysis which included studies before February 2019 to evaluate the influence of paclitaxel-coated balloon angioplasty for patients with FPA diseases and they found no evidence on the increased risk of the two-year all-cause mortality after DCB. Based on individual patient-level data from two single-arm trials and two RCTs, Schneider et al. [78] demonstrated the safety of the use of paclitaxel-coated balloons to treat FPA diseases. An updated meta-analysis conducted by Dinh et al. [79] identified 34 RCTs before December 2020 and reported no association between the increased risk of all-cause mortality and the implementation of paclitaxel devices. Mathlouthi et al. [80] performed a retrospective study on patients receiving paclitaxel-eluting stents and claimed no difference in the 2-year all-cause mortality in the real-world setting. Nevertheless, they observed a significantly higher risk of death among patients who required longer stents and higher paclitaxel doses. In addition, several retrospective cohort studies using large real-world datasets [81–84] were conducted to investigate safety outcomes of paclitaxel-coated devices while the late mortality signal could not be detected in any of these studies.

In conclusion, we observed no association between increased risk of death and the use of paclitaxel while with a longer follow-up period, the pooled results yielded a higher estimate of RR for paclitaxel versus control. On the other hand, paclitaxel DCBs and DESs have shown superiority over the standard uncoated endovascular therapies in reducing restenosis, target lesion revascularization and improving the quality of life. Considering the trustworthy clinical effectiveness and potential long-term risk, the risk-benefit profiles of paclitaxel DCBs and DESs are still of uncertainty. In real clinical practice, it is suggested to assess whether the treatment benefit of using paclitaxel in balloons and stents could outweigh the risk of mortality for each individual patient [8, 9, 69, 81].

As an update of the work by Katsanos et al. [7], this study has several limitations. The entire analysis was performed on the aggregate-level data. In the presence of censoring, survival analysis on time-to-event observations would be more appropriate, while for most of the included RCTs we had no access to individual patient data and the summarized binary data were used instead. When counting the number of patients, we excluded patients who were lost to follow-up to alleviate potential bias. Without the original patient-level data, we were unable to investigate the relationship between patient characteristics and survival or explore undetected sources of clinical heterogeneity. Most of the included RCTs focused on the evaluation of benefits of paclitaxel-coated devices (e.g., patency, late lumen loss, restenosis) and mortality was not the primary endpoint used for the trial design.

In the cumulative meta-analysis by years of publications, we observed a decreasing trend of RR for the two- and five-year all-cause mortality, indicating that paclitaxel-coated/eluting devices implemented in recent years were less toxic to patients with PAD. The improvement of products using paclitaxel might make substantial contributions to the treatment of lesions in the femoropopliteal arteries. However, in this study we did not consider this issue since there were few studies investigating the efficacy and safety of a specific paclitaxel device and the corresponding subgroup analysis could not provide convincing evidence. We focused on the use of paclitaxel-coated/eluting devices in FPA and excluded RCTs for infrapopliteal artery diseases. The safety of paclitaxel exposure in below-the-knee arteries is also a controversial topic [85] and several meta-analyses have been conducted [86–88]. Another limitation of our study is that we only considered a single safety endpoint of all-cause mortality and did not examine the association between the use of paclitaxel and other safety outcomes, e.g., thrombosis, allergy and major amputation of the target limb.

## Conclusion

This systematic review and summary-level meta-analysis showed that the use of paclitaxel DCBs and DESs was not associated with the increased short- or long-term mortality. Concerning well-proven clinical effectiveness of paclitaxel devices in the femoropopliteal arteries, further investigations including more RCTs with longer follow-up periods and individual patient-level data are warranted to shed more light on the risk-benefit profiles of paclitaxel usage in PAD patients.

## Supporting information

S1 Fig Risk of bias assessment. (TIF)

S2 Fig Funnel plots at (a) one year; (b) two years; (c) five years. (TIF)

S1 Table Search strategy of online databases. (DOCX)

S2 Table Detailed demographic statistics of included randomized controlled trials. (DOCX)

S3 Table Detailed angiographic statistics of included randomized controlled trials. (DOCX)

S4 Table All-cause mortality at one, two and five years of included randomized controlled trials. (DOCX)

S5 Table Sensitivity analyses of all-cause mortality. Risk ratio (95% CI) for frequentist methods and odds ratio (95% equal-tailed CrI) for Bayesian methods.
(DOCX)

S6 Table Cumulative meta-analysis by year of publication. (DOCX)

S7 Table Egger’s test of publication bias. (DOCX)

S1 Checklist (DOCX)

## References

[1] Peach G, Griffin M, Jones KG, Thompson MM, Hinchliffe RJ. Diagnosis and management of peripheral arterial disease. BMJ. 2012;345:e5208. Epub 2012/08/16. doi: 10.1136/bmj.e5208 . 22893640

[2] Layden J, Michaels J, Bermingham S, Higgins B, Guideline Development G. Diagnosis and management of lower limb peripheral arterial disease: summary of NICE guidance. BMJ. 2012;345:e4947. Epub 2012/08/10. doi: 10.1136/bmj.e4947 . 22875949

[3] Kasapis C, Henke PK, Chetcuti SJ, Koenig GC, Rectenwald JE, Krishnamurthy VN, et al. Routine stent implantation vs. percutaneous transluminal angioplasty in femoropopliteal artery disease: a meta-analysis of randomized controlled trials. Eur Heart J. 2009;30(1):44–55. Epub 2008/11/26. doi: 10.1093/eurheartj/ehn514 . 19028778

[4] Cassese S, Byrne RA, Ott I, Ndrepepa G, Nerad M, Kastrati A, et al. Paclitaxel-coated versus uncoated balloon angioplasty reduces target lesion revascularization in patients with femoropopliteal arterial disease: a meta-analysis of randomized trials. Circ Cardiovasc Interv. 2012;5(4):582–9. Epub 2012/08/02. doi: 10.1161/CIRCINTERVENTIONS.112.969972 . 22851526

[5] Klumb C, Lehmann T, Aschenbach R, Eckardt N, Teichgraber U. Benefit and risk from paclitaxel-coated balloon angioplasty for the treatment of femoropopliteal artery disease: A systematic review and meta-analysis of randomised controlled trials. EClinicalMedicine. 2019;16:42–50. Epub 2019/12/14. doi: 10.1016/j.eclinm.2019.09.004 31832619 PMC6890981

[6] Laird JA, Schneider PA, Jaff MR, Brodmann M, Zeller T, Metzger DC, et al. Long-Term Clinical Effectiveness of a Drug-Coated Balloon for the Treatment of Femoropopliteal Lesions. Circ Cardiovasc Interv. 2019;12(6):e007702. Epub 2019/06/15. doi: 10.1161/CIRCINTERVENTIONS.118.007702 31195825 PMC6636795

[7] Katsanos K, Spiliopoulos S, Kitrou P, Krokidis M, Karnabatidis D. Risk of Death Following Application of Paclitaxel-Coated Balloons and Stents in the Femoropopliteal Artery of the Leg: A Systematic Review and Meta-Analysis of Randomized Controlled Trials. J Am Heart Assoc. 2018;7(24):e011245. Epub 2018/12/19. doi: 10.1161/JAHA.118.011245 30561254 PMC6405619

[8] FDA. Treatment of Peripheral Arterial Disease with Paclitaxel-Coated Balloons and Paclitaxel-Eluting Stents Potentially Associated with Increased Mortality—Letter to Health Care Providers. FDA; January 17, 2019.

[9] FDA. UPDATE: Treatment of Peripheral Arterial Disease with Paclitaxel-Coated Balloons and Paclitaxel-Eluting Stents Potentially Associated with Increased Mortality—Letter to Health Care Providers. In: FDA, editor. March 15, 2019.

[10] Nordanstig J, James S, Andersson M, Andersson M, Danielsson P, Gillgren P, et al. Mortality with Paclitaxel-Coated Devices in Peripheral Artery Disease. N Engl J Med. 2020;383(26):2538–46. Epub 2020/12/10. doi: 10.1056/NEJMoa2005206 . 33296560

[11] Hunt BD, Popplewell MA, Davies H, Meecham L, Jarrett H, Bate G, et al. BAlloon versus Stenting in severe Ischaemia of the Leg-3 (BASIL-3): study protocol for a randomised controlled trial. Trials. 2017;18(1):224. Epub 2017/05/21. doi: 10.1186/s13063-017-1968-6 28526046 PMC5438558

[12] Agency MHpR. Recommendations from the independent Expert Advisory Group on the use of Paclitaxel Drug Coated Balloons (DCBs) and Drug Eluting Stents (DESs) to the MHRA. In: Agency MHpR, editor. June 3, 2019.

[13] Beckman JA, White CJ. Paclitaxel-Coated Balloons and Eluting Stents: Is There a Mortality Risk in Patients With Peripheral Artery Disease? Circulation. 2019;140(16):1342–51. doi: 10.1161/CIRCULATIONAHA.119.041099 31177820

[14] Page MJ, McKenzie JE, Bossuyt PM, Boutron I, Hoffmann TC, Mulrow CD, et al. The PRISMA 2020 statement: an updated guideline for reporting systematic reviews. BMJ. 2021;372:n71. Epub 2021/03/31. doi: 10.1136/bmj.n71 . 33782057 PMC8005924

[15] Sterne JAC, Savovic J, Page MJ, Elbers RG, Blencowe NS, Boutron I, et al. RoB 2: a revised tool for assessing risk of bias in randomised trials. BMJ. 2019;366:l4898. Epub 2019/08/30. doi: 10.1136/bmj.l4898 . 31462531

[16] LRJP D.B. Summing Up: The Science of Reviewing Research. Cambridge: Harvard University Press; 1984.

[17] Egger M, Davey Smith G, Schneider M, Minder C. Bias in meta-analysis detected by a simple, graphical test. BMJ. 1997;315(7109):629–34. Epub 1997/10/06. doi: 10.1136/bmj.315.7109.629 9310563 PMC2127453

[18] Varu VN, Hogg ME, Kibbe MR. Critical limb ischemia. Journal of Vascular Surgery. 2010;51(1):230–41. 10.1016/j.jvs.2009.08.073. 20117502

[19] Sachar R, Soga Y, Ansari MM, Kozuki A, Lopez L, Brodmann M, et al. 1-Year Results From the RANGER II SFA Randomized Trial of the Ranger Drug-Coated Balloon. JACC: Cardiovascular Interventions. 2021;14(10):1123–33. doi: 10.1016/j.jcin.2021.03.021 34016410

[20] Brodmann M, editor 2-Year Results of the RANGER II SFA Randomized Trial. LINC symposium; June 2022.

[21] Steiner S, Willfort-Ehringer A, Sievert H, Geist V, Lichtenberg M, Del Giudice C, et al. 12-Month Results From the First-in-Human Randomized Study of the Ranger Paclitaxel-Coated Balloon for Femoropopliteal Treatment. JACC Cardiovasc Interv. 2018;11(10):934–41. Epub 2018/05/08. doi: 10.1016/j.jcin.2018.01.276 . 29730375

[22] Jia X, Zhang J, Zhuang B, Fu W, Wu D, Wang F, et al. Acotec Drug-Coated Balloon Catheter: Randomized, Multicenter, Controlled Clinical Study in Femoropopliteal Arteries: Evidence From the AcoArt I Trial. JACC Cardiovasc Interv. 2016;9(18):1941–9. Epub 2016/09/24. doi: 10.1016/j.jcin.2016.06.055 . 27659572

[23] Tepe G, Gogebakan O, Redlich U, Tautenhahn J, Ricke J, Halloul Z, et al. Angiographic and Clinical Outcomes After Treatment of Femoro-Popliteal Lesions with a Novel Paclitaxel-Matrix-Coated Balloon Catheter. Cardiovasc Intervent Radiol. 2017;40(10):1535–44. Epub 2017/07/01. doi: 10.1007/s00270-017-1713-2 . 28660441

[24] Tepe G, Schnorr B, Albrecht T, Brechtel K, Claussen CD, Scheller B, et al. Angioplasty of femoral-popliteal arteries with drug-coated balloons: 5-year follow-up of the THUNDER trial. JACC Cardiovasc Interv. 2015;8(1 Pt A):102–8. Epub 2015/01/27. doi: 10.1016/j.jcin.2014.07.023 . 25616822

[25] Falkowski A, Bogacki H, Szemitko M. Assessment of Mortality and Factors Affecting Outcome of Use of Paclitaxel-Coated Stents and Bare Metal Stents in Femoropopliteal PAD. Journal of Clinical Medicine. 2020;9(7):2221. doi: 10.3390/jcm9072221 32668743 PMC7408889

[26] FDA. Circulatory System Devices Panel June 19, 2019 FDA Executive Summary. In: FDA, editor. 2019.

[27] Fanelli F, Cannavale A, Corona M, Lucatelli P, Wlderk A, Salvatori FM. The "DEBELLUM"—lower limb multilevel treatment with drug eluting balloon—randomized trial: 1-year results. J Cardiovasc Surg (Torino). 2014;55(2):207–16. Epub 2014/03/29. . 24670828

[28] de Boer SW, de Vries J, Werson DA, Fioole B, Vroegindeweij D, Vos JA, et al. Drug coated balloon supported Supera stent versus Supera stent in intermediate and long-segment lesions of the superficial femoral artery: 2-year results of the RAPID Trial. J Cardiovasc Surg (Torino). 2019;60(6):679–85. Epub 2019/10/12. doi: 10.23736/S0021-9509.19.11109-3 . 31603295

[29] Xu Y, Jia X, Zhang J, Zhuang B, Fu W, Wu D, et al. Drug-Coated Balloon Angioplasty Compared With Uncoated Balloons in the Treatment of 200 Chinese Patients With Severe Femoropopliteal Lesions: 24-Month Results of AcoArt I. JACC Cardiovasc Interv. 2018;11(23):2347–53. Epub 2018/11/19. doi: 10.1016/j.jcin.2018.07.041 . 30448170

[30] Teichgraber U, Lehmann T, Aschenbach R, Scheinert D, Zeller T, Brechtel K, et al. Drug-coated Balloon Angioplasty of Femoropopliteal Lesions Maintained Superior Efficacy over Conventional Balloon: 2-year Results of the Randomized EffPac Trial. Radiology. 2020;295(2):478–87. Epub 2020/03/04. doi: 10.1148/radiol.2020191619 . 32125256

[31] Krankenberg H, Tubler T, Ingwersen M, Schluter M, Scheinert D, Blessing E, et al. Drug-Coated Balloon Versus Standard Balloon for Superficial Femoral Artery In-Stent Restenosis: The Randomized Femoral Artery In-Stent Restenosis (FAIR) Trial. Circulation. 2015;132(23):2230–6. Epub 2015/10/09. doi: 10.1161/CIRCULATIONAHA.115.017364 . 26446728

[32] Tepe G, Laird J, Schneider P, Brodmann M, Krishnan P, Micari A, et al. Drug-coated balloon versus standard percutaneous transluminal angioplasty for the treatment of superficial femoral and popliteal peripheral artery disease: 12-month results from the IN.PACT SFA randomized trial. Circulation. 2015;131(5):495–502. Epub 2014/12/05. doi: 10.1161/CIRCULATIONAHA.114.011004 25472980 PMC4323569

[33] Iida O, Soga Y, Urasawa K, Saito S, Jaff MR, Wang H, et al. Drug-coated balloon versus uncoated percutaneous transluminal angioplasty for the treatment of atherosclerotic lesions in the superficial femoral and proximal popliteal artery: 2-year results of the MDT-2113 SFA Japan randomized trial. Catheter Cardiovasc Interv. 2019;93(4):664–72. Epub 2019/02/13. doi: 10.1002/ccd.28048 30747489 PMC6594002

[34] Iida O, Soga Y, Urasawa K, Saito S, Jaff MR, Wang H, et al. Drug-Coated Balloon vs Standard Percutaneous Transluminal Angioplasty for the Treatment of Atherosclerotic Lesions in the Superficial Femoral and Proximal Popliteal Arteries: One-Year Results of the MDT-2113 SFA Japan Randomized Trial. J Endovasc Ther. 2018;25(1):109–17. Epub 2017/12/22. doi: 10.1177/1526602817745565 29264999 PMC5774613

[35] Bjorkman P, Kokkonen T, Alback A, Venermo M. Drug-Coated versus Plain Balloon Angioplasty in Bypass Vein Grafts (the DRECOREST I-Study). Ann Vasc Surg. 2019;55:36–44. Epub 2018/08/10. doi: 10.1016/j.avsg.2018.04.042 . 30092443

[36] Liistro F, Grotti S, Porto I, Angioli P, Ricci L, Ducci K, et al. Drug-eluting balloon in peripheral intervention for the superficial femoral artery: the DEBATE-SFA randomized trial (drug eluting balloon in peripheral intervention for the superficial femoral artery). JACC Cardiovasc Interv. 2013;6(12):1295–302. Epub 2013/11/19. doi: 10.1016/j.jcin.2013.07.010 . 24239203

[37] Björkman P, Auvinen T, Hakovirta H, Romsi P, Turtiainen J, Manninen H, et al. Drug-Eluting Stent Shows Similar Patency Results as Prosthetic Bypass in Patients with Femoropopliteal Occlusion in a Randomized Trial. Annals of Vascular Surgery. 2018;53:165–70. 10.1016/j.avsg.2018.04.014. 29886215

[38] Miura T, Miyashita Y, Soga Y, Hozawa K, Doijiri T, Ikeda U, et al. Drug-Eluting Versus Bare-Metal Stent Implantation With or Without Cilostazol in the Treatment of the Superficial Femoral Artery. Circ Cardiovasc Interv. 2018;11(8):e006564. Epub 2018/10/26. doi: 10.1161/CIRCINTERVENTIONS.118.006564 . 30354784

[39] Laird JR, Schneider PA, Tepe G, Brodmann M, Zeller T, Metzger C, et al. Durability of Treatment Effect Using a Drug-Coated Balloon for Femoropopliteal Lesions: 24-Month Results of IN.PACT SFA. J Am Coll Cardiol. 2015;66(21):2329–38. Epub 2015/10/20. doi: 10.1016/j.jacc.2015.09.063 . 26476467

[40] Dake MD, Ansel GM, Jaff MR, Ohki T, Saxon RR, Smouse HB, et al. Durable Clinical Effectiveness With Paclitaxel-Eluting Stents in the Femoropopliteal Artery: 5-Year Results of the Zilver PTX Randomized Trial. Circulation. 2016;133(15):1472–83; discussion 83. Epub 2016/03/13. doi: 10.1161/CIRCULATIONAHA.115.016900 26969758 PMC4823823

[41] Teichgraber U, Lehmann T, Aschenbach R, Scheinert D, Zeller T, Brechtel K, et al. Efficacy and safety of a novel paclitaxel-nano-coated balloon for femoropopliteal angioplasty: one-year results of the EffPac trial. EuroIntervention. 2020;15(18):e1633–e40. Epub 2019/11/07. doi: 10.4244/EIJ-D-19-00292 . 31687933

[42] Gouëffic Y, editor EMINENT: Randomized Trial of Eluvia DES vs Bare Metal Stents 12-month Effectiveness, Safety and Subgroup Analysis. LINC symposium; June 2022.

[43] Brodmann M, editor Final Long-Term Mortality Results of Paclitaxel-Coated DCBs from the ILLUMENATE RCTs 5-Year Data from EU RCT and Pivotal studies. LINC symposium; January 2021.

[44] Werk M, Langner S, Reinkensmeier B, Boettcher H-F, Tepe G, Dietz U, et al. Inhibition of Restenosis in Femoropopliteal Arteries. Circulation. 2008;118(13):1358–65. doi: 10.1161/CIRCULATIONAHA.107.735985 18779447

[45] Ott I, Cassese S, Groha P, Steppich B, Voll F, Hadamitzky M, et al. ISAR-PEBIS (Paclitaxel-Eluting Balloon Versus Conventional Balloon Angioplasty for In-Stent Restenosis of Superficial Femoral Artery): A Randomized Trial. J Am Heart Assoc. 2017;6(7). Epub 2017/07/27. doi: 10.1161/JAHA.117.006321 28743787 PMC5586321

[46] Scheinert D, Duda S, Zeller T, Krankenberg H, Ricke J, Bosiers M, et al. The LEVANT I (Lutonix paclitaxel-coated balloon for the prevention of femoropopliteal restenosis) trial for femoropopliteal revascularization: first-in-human randomized trial of low-dose drug-coated balloon versus uncoated balloon angioplasty. JACC Cardiovasc Interv. 2014;7(1):10–9. Epub 2014/01/25. doi: 10.1016/j.jcin.2013.05.022 . 24456716

[47] Tepe G, Zeller T, Albrecht T, Heller S, Schwarzwalder U, Beregi JP, et al. Local delivery of paclitaxel to inhibit restenosis during angioplasty of the leg. N Engl J Med. 2008;358(7):689–99. Epub 2008/02/15. doi: 10.1056/NEJMoa0706356 . 18272892

[48] Xu Y, Liu J, Zhang J, Zhuang B, Jia X, Fu W, et al. Long-term safety and efficacy of angioplasty of femoropopliteal artery disease with drug-coated balloons from the AcoArt I trial. J Vasc Surg. 2021. Epub 2021/02/19. doi: 10.1016/j.jvs.2021.01.041 . 33600928

[49] Schroeder H, Werner M, Meyer DR, Reimer P, Kruger K, Jaff MR, et al. Low-Dose Paclitaxel-Coated Versus Uncoated Percutaneous Transluminal Balloon Angioplasty for Femoropopliteal Peripheral Artery Disease: One-Year Results of the ILLUMENATE European Randomized Clinical Trial (Randomized Trial of a Novel Paclitaxel-Coated Percutaneous Angioplasty Balloon). Circulation. 2017;135(23):2227–36. Epub 2017/04/21. doi: 10.1161/CIRCULATIONAHA.116.026493 28424223 PMC5459585

[50] Rosenfield K, editor The LUTONIX DCB Program Safety Track Record and Other Considerations. Cardiovascular Research Technologies Conference; May 2019.

[51] Nowakowski P, Uchto W, Hrycek E, Kachel M, Ludyga T, Polczyk F, et al. Microcrystalline paclitaxel-coated balloon for revascularization of femoropopliteal artery disease: Three-year outcomes of the randomized BIOPAC trial. Vascular Medicine. 2021;26(4):401–8. doi: 10.1177/1358863X20988360 . 33686879

[52] Ni L, Ye W, Zhang L, Jin X, Shu C, Jiang J-s, et al. A Multicenter Randomized Trial Assessing ZENFlow Carrier-Free Drug-Coated Balloon for the Treatment of Femoropopliteal Artery Lesions. Frontiers in Cardiovascular Medicine. 2022;9. doi: 10.3389/fcvm.2022.821672 35391838 PMC8982076

[53] Teichgräber U, editor New safety and effectiveness data of Luminor DCB at 5 years: the EffPAC trial. LINC symposium; June 2022.

[54] Lyden SP, Faries PL, Niazi KAK, Sachar R, Jain A, Brodmann M, et al. No Mortality Signal With Stellarex Low-Dose Paclitaxel DCB: ILLUMENATE Pivotal 4-Year Outcomes. Journal of Endovascular Therapy. 0(0):15266028211068769. doi: 10.1177/15266028211068769 . 35000470

[55] Liao C-J, Song S-H, Li T, Zhang YZ, Wang-de. Orchid drug-coated balloon versus standard percutaneous transluminal angioplasty for the treatment of femoropopliteal artery disease: 12-month result of the randomized controlled trial. Vascular. 2022;30(3):448–54. doi: 10.1177/17085381211013968 . 34024196

[56] Tepe G, Schroeder H, Albrecht T, Reimer P, Diehm N, Baeriswyl JL, et al. Paclitaxel-Coated Balloon vs Uncoated Balloon Angioplasty for Treatment of In-Stent Restenosis in the Superficial Femoral and Popliteal Arteries: The COPA CABANA Trial. J Endovasc Ther. 2020;27(2):276–86. Epub 2020/02/26. doi: 10.1177/1526602820907917 . 32096451

[57] Werk M, Albrecht T, Meyer DR, Ahmed MN, Behne A, Dietz U, et al. Paclitaxel-coated balloons reduce restenosis after femoro-popliteal angioplasty: evidence from the randomized PACIFIER trial. Circ Cardiovasc Interv. 2012;5(6):831–40. Epub 2012/11/30. doi: 10.1161/CIRCINTERVENTIONS.112.971630 . 23192918

[58] Kinstner CM, Lammer J, Willfort-Ehringer A, Matzek W, Gschwandtner M, Javor D, et al. Paclitaxel-Eluting Balloon Versus Standard Balloon Angioplasty in In-Stent Restenosis of the Superficial Femoral and Proximal Popliteal Artery: 1-Year Results of the PACUBA Trial. JACC Cardiovasc Interv. 2016;9(13):1386–92. Epub 2016/07/09. doi: 10.1016/j.jcin.2016.04.012 . 27388828

[59] Dake MD, Ansel GM, Jaff MR, Ohki T, Saxon RR, Smouse HB, et al. Paclitaxel-eluting stents show superiority to balloon angioplasty and bare metal stents in femoropopliteal disease: twelve-month Zilver PTX randomized study results. Circ Cardiovasc Interv. 2011;4(5):495–504. Epub 2011/09/29. doi: 10.1161/CIRCINTERVENTIONS.111.962324 . 21953370

[60] Scheinert D, Schulte KL, Zeller T, Lammer J, Tepe G. Paclitaxel-releasing balloon in femoropopliteal lesions using a BTHC excipient: twelve-month results from the BIOLUX P-I randomized trial. J Endovasc Ther. 2015;22(1):14–21. Epub 2015/03/17. doi: 10.1177/1526602814564383 . 25775674

[61] Goueffic Y, Sauguet A, Desgranges P, Feugier P, Rosset E, Ducasse E, et al. A Polymer-Free Paclitaxel-Eluting Stent Versus a Bare-Metal Stent for De Novo Femoropopliteal Lesions: The BATTLE Trial. JACC Cardiovasc Interv. 2020;13(4):447–57. Epub 2020/02/23. doi: 10.1016/j.jcin.2019.12.028 . 32081238

[62] Ott I, Cassese S, Groha P, Steppich B, Hadamitzky M, Ibrahim T, et al. Randomized Comparison of Paclitaxel-Eluting Balloon and Stenting Versus Plain Balloon Plus Stenting Versus Directional Atherectomy for Femoral Artery Disease (ISAR-STATH). Circulation. 2017;135(23):2218–26. Epub 2017/04/21. doi: 10.1161/CIRCULATIONAHA.116.025329 . 28424222

[63] Liao CJ, Song SH, Li T, Zhang Y, Zhang WD. Randomized controlled trial of orchid drug-coated balloon versus standard percutaneous transluminal angioplasty for treatment of femoropopliteal artery in-stent restenosis. Int Angiol. 2019;38(5):365–71. Epub 2019/10/01. doi: 10.23736/S0392-9590.19.04243-3 . 31566320

[64] Tacke J, Muller-Hulsbeck S, Schroder H, Lammer J, Schurmann K, Gross-Fengels W, et al. The Randomized Freeway Stent Study: Drug-Eluting Balloons Outperform Standard Balloon Angioplasty for Postdilatation of Nitinol Stents in the SFA and PI Segment. Cardiovasc Intervent Radiol. 2019;42(11):1513–21. Epub 2019/08/23. doi: 10.1007/s00270-019-02309-3 31432220 PMC6775030

[65] Ye W, Zhang X, Dai X, Huang X, Liu Z, Jiang Me, et al. Reewarm^™^ PTX drug-coated balloon in the treatment of femoropopliteal artery disease: A multi-center, randomized controlled trial in China. International Journal of Cardiology. 2021;326:164–9. 10.1016/j.ijcard.2020.10.060. 33127414

[66] Ouriel K, Adelman MA, Rosenfield K, Scheinert D, Brodmann M, Pena C, et al. Safety of Paclitaxel-Coated Balloon Angioplasty for Femoropopliteal Peripheral Artery Disease. JACC Cardiovasc Interv. 2019;12(24):2515–24. Epub 2019/10/03. doi: 10.1016/j.jcin.2019.08.025 . 31575518

[67] de Boer SW, van den Heuvel DAF, de Vries-Werson DAB, Vos JA, Fioole B, Vroegindeweij D, et al. Short-term Results of the RAPID Randomized Trial of the Legflow Paclitaxel-Eluting Balloon With Supera Stenting vs Supera Stenting Alone for the Treatment of Intermediate and Long Superficial Femoral Artery Lesions. J Endovasc Ther. 2017;24(6):783–92. Epub 2017/08/11. doi: 10.1177/1526602817725062 . 28795638

[68] Krishnan P, Faries P, Niazi K, Jain A, Sachar R, Bachinsky WB, et al. Stellarex Drug-Coated Balloon for Treatment of Femoropopliteal Disease: Twelve-Month Outcomes From the Randomized ILLUMENATE Pivotal and Pharmacokinetic Studies. Circulation. 2017;136(12):1102–13. Epub 2017/07/22. doi: 10.1161/CIRCULATIONAHA.117.028893 28729250 PMC5598919

[69] FDA. Summary of safety and effectiveness data (SSED, P190019B) In: FDA, editor.: FDA; 2019.

[70] Brodmann M, Werner M, Meyer DR, Reimer P, Kruger K, Granada JF, et al. Sustainable Antirestenosis Effect With a Low-Dose Drug-Coated Balloon: The ILLUMENATE European Randomized Clinical Trial 2-Year Results. JACC Cardiovasc Interv. 2018;11(23):2357–64. Epub 2018/12/14. doi: 10.1016/j.jcin.2018.08.034 . 30522663

[71] Dake MD, Ansel GM, Jaff MR, Ohki T, Saxon RR, Smouse HB, et al. Sustained safety and effectiveness of paclitaxel-eluting stents for femoropopliteal lesions: 2-year follow-up from the Zilver PTX randomized and single-arm clinical studies. J Am Coll Cardiol. 2013;61(24):2417–27. Epub 2013/04/16. doi: 10.1016/j.jacc.2013.03.034 . 23583245

[72] Rosenfield K, Jaff MR, White CJ, Rocha-Singh K, Mena-Hurtado C, Metzger DC, et al. Trial of a Paclitaxel-Coated Balloon for Femoropopliteal Artery Disease. N Engl J Med. 2015;373(2):145–53. Epub 2015/06/25. doi: 10.1056/NEJMoa1406235 . 26106946

[73] Zhang B, Yang M, He T, Li X, Gu J, Zhang X, et al. Twelve-Month Results From the First-in-China Prospective, Multi-Center, Randomized, Controlled Study of the FREEWAY Paclitaxel-Coated Balloon for Femoropopliteal Treatment. Frontiers in Cardiovascular Medicine. 2021;8. doi: 10.3389/fcvm.2021.686267 34568443 PMC8460758

[74] Albrecht T, Waliszewski M, Roca C, Redlich U, Tautenhahn J, Pech M, et al. Two-Year Clinical Outcomes of the CONSEQUENT Trial: Can Femoropopliteal Lesions be Treated with Sustainable Clinical Results that are Economically Sound? Cardiovasc Intervent Radiol. 2018;41(7):1008–14. Epub 2018/03/29. doi: 10.1007/s00270-018-1940-1 . 29589098

[75] Albrecht T, Schnorr B, Kutschera M, Waliszewski MW. Two-Year Mortality After Angioplasty of the Femoro-Popliteal Artery with Uncoated Balloons and Paclitaxel-Coated Balloons-A Pooled Analysis of Four Randomized Controlled Multicenter Trials. Cardiovasc Intervent Radiol. 2019;42(7):949–55. Epub 2019/03/08. doi: 10.1007/s00270-019-02194-w . 30843092

[76] Bittl JA, He Y, Baber U, Feldman RL, von Mering GO, Kaul S. Bayes Factor Meta-Analysis of the Mortality Claim for Peripheral Paclitaxel-Eluting Devices. JACC Cardiovasc Interv. 2019;12(24):2528–37. Epub 2019/12/21. doi: 10.1016/j.jcin.2019.09.028 . 31857023

[77] Rocha-Singh KJ, Duval S, Jaff MR, Schneider PA, Ansel GM, Lyden SP, et al. Mortality and Paclitaxel-Coated Devices: An Individual Patient Data Meta-Analysis. Circulation. 2020;141(23):1859–69. Epub 2020/05/07. doi: 10.1161/CIRCULATIONAHA.119.044697 32370548 PMC8029645

[78] Schneider PA, Laird JR, Doros G, Gao Q, Ansel G, Brodmann M, et al. Mortality Not Correlated With Paclitaxel Exposure. Journal of the American College of Cardiology. 2019;73(20):2550–63. doi: 10.1016/j.jacc.2019.01.013 30690141

[79] Dinh K, Limmer AM, Chen AZL, Thomas SD, Holden A, Schneider PA, et al. Mortality Rates After Paclitaxel-Coated Device Use in Patients With Occlusive Femoropopliteal Disease: An Updated Systematic Review and Meta-Analysis of Randomized Controlled Trials. Journal of Endovascular Therapy. 2021;28(5):755–77. doi: 10.1177/15266028211023505 . 34106028

[80] Mathlouthi A, Yei KS, Naazie I, Bertges DJ, Malas MB. Increased mortality with paclitaxel-eluting stents is driven by lesion length. Journal of Vascular Surgery. 2021;73(2):548–53.e2. doi: 10.1016/j.jvs.2020.05.061 32615286

[81] Bertges DJ, Sedrakyan A, Sun T, Eslami MH, Schermerhorn M, Goodney PP, et al. Mortality After Paclitaxel Coated Balloon Angioplasty and Stenting of Superficial Femoral and Popliteal Artery in the Vascular Quality Initiative. Circ Cardiovasc Interv. 2020;13(2):e008528. Epub 2020/02/19. doi: 10.1161/CIRCINTERVENTIONS.119.008528 . 32069110

[82] Behrendt CA, Sedrakyan A, Peters F, Kreutzburg T, Schermerhorn M, Bertges DJ, et al. Editor’s Choice—Long Term Survival after Femoropopliteal Artery Revascularisation with Paclitaxel Coated Devices: A Propensity Score Matched Cohort Analysis. Eur J Vasc Endovasc Surg. 2020;59(4):587–96. Epub 2020/01/14. doi: 10.1016/j.ejvs.2019.12.034 . 31926836

[83] Secemsky EA, Kundi H, Weinberg I, Schermerhorn M, Beckman JA, Parikh SA, et al. Drug-Eluting Stent Implantation and Long-Term Survival Following Peripheral Artery Revascularization. J Am Coll Cardiol. 2019;73(20):2636–8. Epub 2019/03/05. doi: 10.1016/j.jacc.2019.02.020 . 30831175

[84] Secemsky EA, Kundi H, Weinberg I, Jaff MR, Krawisz A, Parikh SA, et al. Association of Survival With Femoropopliteal Artery Revascularization With Drug-Coated Devices. JAMA Cardiol. 2019;4(4):332–40. Epub 2019/02/13. doi: 10.1001/jamacardio.2019.0325 30747949 PMC6484791

[85] Katsanos K, Spiliopoulos S. Safety of Paclitaxel-Eluting Stents in Below-the-Knee Arteries for Critical Limb Ischemia Treatment: the Devil is in the Details. CardioVascular and Interventional Radiology. 2020;43(12):1889–90. doi: 10.1007/s00270-020-02651-x 32965583

[86] Ipema J, Huizing E, Schreve MA, de Vries J-PPM, Ünlü Ç. Editor’s Choice–Drug Coated Balloon Angioplasty vs. Standard Percutaneous Transluminal Angioplasty in Below the Knee Peripheral Arterial Disease: A Systematic Review and Meta-Analysis. European Journal of Vascular and Endovascular Surgery. 2020;59(2):265–75. 10.1016/j.ejvs.2019.10.002. 31889657

[87] Katsanos K, Spiliopoulos S, Kitrou P, Krokidis M, Paraskevopoulos I, Karnabatidis D. Risk of Death and Amputation with Use of Paclitaxel-Coated Balloons in the Infrapopliteal Arteries for Treatment of Critical Limb Ischemia: A Systematic Review and Meta-Analysis of Randomized Controlled Trials. Journal of Vascular and Interventional Radiology. 2020;31(2):202–12. 10.1016/j.jvir.2019.11.015. 31954604

[88] Cai H, Dong J, Ye Y, Song Q, Lu S. Safety and Efficacy of Drug-Coated Balloon in the Treatment of Below-The-Knee Artery: A Meta-analysis. Journal of Surgical Research. 2022;278:303–16. 10.1016/j.jss.2022.04.055. 35660302

