# Supplementary figures and images for: Safety of paclitaxel-coated devices in the femoropopliteal arteries: A systematic review and meta-analysis
Source: PLoS One. 2022 Oct 13;17(10):e0275888. doi: 10.1371/journal.pone.0275888 (PMC9560511; doi:10.1371/journal.pone.0275888)

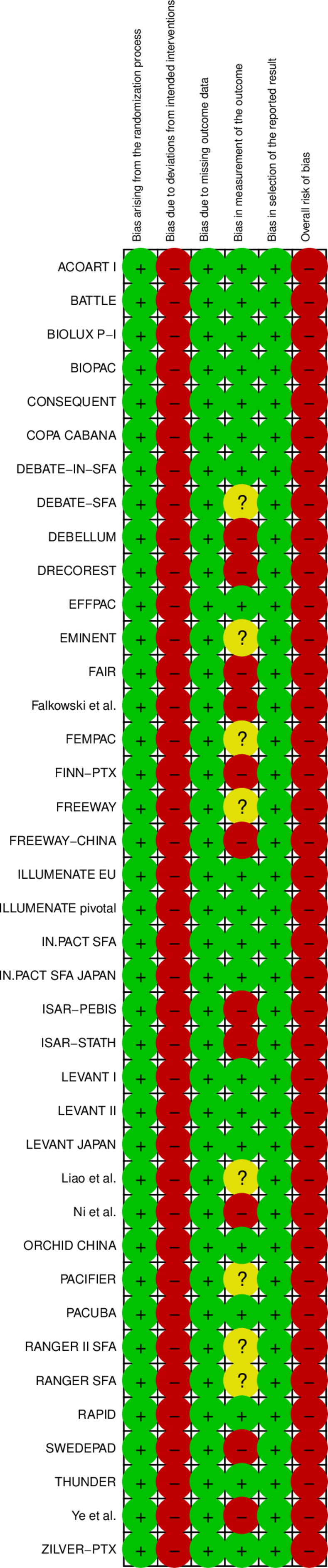

Supplement: S1 Fig — (TIF) [file pone.0275888.s001.tif]

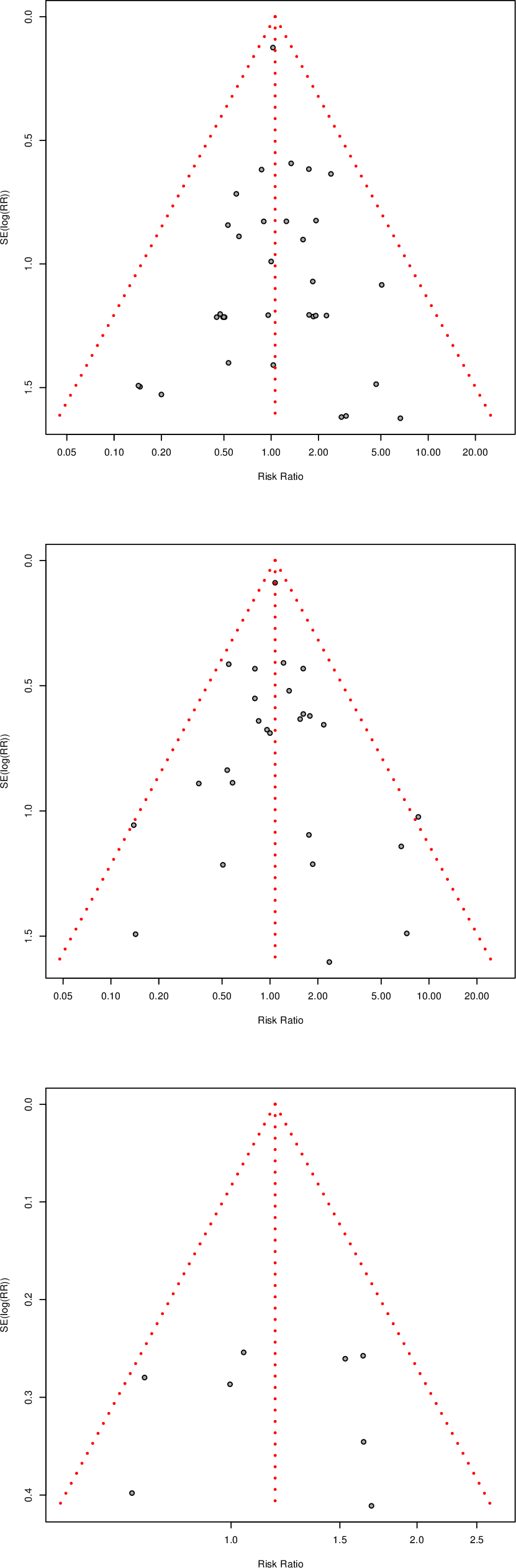

Supplement: S2 Fig — (TIF) [file pone.0275888.s002.tif]
