# Supplementary material for: Safety of paclitaxel-coated devices in the femoropopliteal arteries: A systematic review and meta-analysis
Source: PLoS One. 2022 Oct 13;17(10):e0275888. doi: 10.1371/journal.pone.0275888 (PMC9560511; doi:10.1371/journal.pone.0275888)
Supplement: S1 Table — (DOCX) [file pone.0275888.s003.docx]

**S1 Table. Search Strategy of online databases**

| **Database** | **Search Strategy** |
| --- | --- |
| PubMed (MEDLINE) | (((paclitaxel-eluting[Title/Abstract]) OR (paclitaxel-coated[Title/Abstract]) OR (drug-coated[Title/Abstract]) OR (drug-eluting[Title/Abstract]) OR (DCB[Title/Abstract]) OR (DEB[Title/Abstract]) OR (DCS[Title/Abstract]) OR (DES[Title/Abstract])) AND ((femoral artery[Title/Abstract]) OR (popliteal artery[Title/Abstract]) OR (femoropopliteal artery[Title/Abstract]) OR (late lumen loss[Title/Abstract]) OR (restenosis[Title/Abstract]) OR (target lesion[Title/Abstract]) OR (revascularization[Title/Abstract]) OR (peripheral angioplasty[Title/Abstract])) AND ((Randomized controlled[Title/Abstract]) OR (Randomised controlled[Title/Abstract]) OR (RCT[Title/Abstract]))) AND (("2018/08/01"[Date - Publication] : "3000"[Date - Publication])) |
| Scopus | (TITLE-ABS-KEY(paclitaxel-eluting) OR TITLE-ABS-KEY(paclitaxel-coated) OR TITLE-ABS-KEY(drug-coated) OR TITLE-ABS-KEY(drug-eluting) OR TITLE-ABS-KEY(DCB) OR (DEB) OR TITLE-ABS-KEY(DCS) OR TITLE-ABS-KEY(DES)) AND (TITLE-ABS-KEY(Randomized) OR TITLE-ABS-KEY(Randomised) OR TITLE-ABS-KEY (RCT)) AND (TITLE-ABS-KEY(femoral artery) OR TITLE-ABS-KEY(popliteal artery) OR TITLE-ABS-KEY(femoropopliteal artery) OR TITLE-ABS-KEY(late lumen loss) OR TITLE-ABS-KEY(restenosis) OR TITLE-ABS-KEY(target lesion) OR TITLE-ABS-KEY(revascularization) OR TITLE-ABS-KEY(peripheral angioplasty)) AND PUBYEAR AFT 2018 |
| EMBASE (Ovid) | ("paclitaxel" OR "drug eluting stent" OR "drug-coated balloon" OR "percutaneous transluminal angioplasty" OR "restenosis" OR "femoral artery" OR "popliteal artery" OR "peripheral occlusive artery disease" OR "revascularization" OR "artery lesion" OR "target lesion revascularization") AND "randomized controlled trial" AND (limit 1 to yr="2018") |
