# Supplementary material for: Safety of paclitaxel-coated devices in the femoropopliteal arteries: A systematic review and meta-analysis
Source: PLoS One. 2022 Oct 13;17(10):e0275888. doi: 10.1371/journal.pone.0275888 (PMC9560511; doi:10.1371/journal.pone.0275888)
Supplement: S2 Table — (DOCX) [file pone.0275888.s004.docx]

**S2 Table. Detailed demographic statistics of the 39 included randomized controlled trials.**

| TrialName | T,N | C,N | T,Age | C,Age | T,Male | C,Male | T,Smoker | C,Smoker | T,Hypertension | C,Hypertension |
| --- | --- | --- | --- | --- | --- | --- | --- | --- | --- | --- |
| ZILVER-PTX | 241 | 238 | 67.9±9.6 | 67.7±10.6 | 155 | 152 | 204 | 200 | 210 | 194 |
| THUNDER | 48 | 54 | 69±8 | 68±9 | 31 | 34 | 11 | 12 | 38 | 45 |
| IN.PACT SFA | 220 | 111 | 67.5±9.5 | 68.0±9.2 | 143 | 75 | 85 | 40 | 201 | 98 |
| FEMPAC | 45 | 42 | 67.3 (IQR 63.5–76.4) | 70.2 (IQR 66.2–77.6) | 27 | 25 | 21 | 15 | 35 | 34 |
| LEVANT I | 49 | 52 | 67±8 | 70±10 | 34 | 30 | 15 | 20 | 47 | 45 |
| LEVANT II | 316 | 160 | 67.8±10.0 | 69.0±9.0 | 193 | 107 | 111 | 54 | 282 | 140 |
| ILLUMENATE EU | 222 | 72 | 67±9 | 69±9 | 160 | 49 | 198 | 60 | 173 | 60 |
| CONSEQUENT | 78 | 75 | 68.2 ± 8.5 | 68.0 ± 9.0 | 47 | 57 | 36 | 37 | 60 | 60 |
| ISAR-STATH | 48 | 52 | 69.7±9.4 | 69.2±8 | 33 | 37 | 36 | 34 | 40 | 40 |
| ISAR-PEBIS | 36 | 34 | 70±10 | 68±10 | 24 | 24 | 21 | 24 | 33 | 33 |
| IN.PACT SFA JAPAN | 68 | 32 | 73.3±7.4 | 74.2±6.1 | 50 | 26 | 18 | 10 | NA | NA |
| ACOART I | 100 | 100 | 65.9±9.0 | 65.6±8.6 | 73 | 74 | 29 | 33 | 62 | 72 |
| FINN-PTX | 23 | 18 | 68 (Range 48-88) | 67 (Range 50-84) | 17 | 12 | 9 | 6 | 15 | 15 |
| BATTLE | 86 | 85 | 71±12 | 68±12 | 62 | 62 | 20 | 28 | 59 | 52 |
| DEBATE-IN-SFA | 85 | 85 | 73.1±7.8 | 73.4±8.0 | 60 | 55 | 69 | 64 | 68 | 73 |
| DEBELLUM | 25 | 25 | 67±7 | 67±6 | 19 | 18 | 17 | 14 | 19 | 15 |
| PACIFIER | 44 | 47 | 71±7 | 71±9 | 26 | 30 | 21 | 28 | 29 | 31 |
| FAIR | 62 | 57 | 69±8 | 67±9 | 33 | 49 | 18 | 20 | 52 | 54 |
| BIOLUX P-I | 30 | 30 | 70.1±10.4 | 71.4±10.0 | 17 | 17 | 19 | 22 | 23 | 21 |
| RANGER SFA | 71 | 34 | 68±8 | 67±9 | 53 | 23 | 29 | 17 | 58 | 26 |
| ILLUMENATE pivotal | 200 | 100 | 68.3±10.3 | 69.8±9.8 | 112 | 64 | 168 | 75 | 187 | 94 |
| DEBATE-SFA | 53 | 51 | 74±9 | 76±8 | 50 | 42 | 25 | 28 | 47 | 45 |
| LEVANT JAPAN | 71 | 38 | 72.5±9.8 | 78.2±8.1 | 45 | 46 | 53 | 26 | 60 | 35 |
| RAPID | 80 | 80 | 67.6±7.5 | 67.0±8.0 | 52 | 50 | 40 | 39 | NA | NA |
| EFFPAC | 85 | 86 | 68±8 | 68±9 | 51 | 60 | 34 | 37 | 74 | 73 |
| PACUBA | 35 | 39 | 68.1±9.2 | 68.3±0.4 | 20 | 23 | 17 | 18 | 26 | 27 |
| FREEWAY | 105 | 99 | 64.7±9.4 | 64.3±9.8 | 82 | 76 | 93 | 81 | 79 | 73 |
| DRECOREST | 29 | 28 | 70.4 (Range 45-88) | 72.3 (Range 55-89) | 15 | 17 | 17 | 14 | 23 | 20 |
| SWEDEPAD | 1149 | 1140 | 75.4 (IQR 70.0–82.1) | 75.6 (IQR 69.9–81.6) | 626 | 628 | 708 | 723 | 921 | 914 |
| Falkowski et al. | 126 | 130 | 66.6±7.33 | 64.9±8.10 | 80 | 82 | NA | NA | 102 | 106 |
| COPA CABANA | 47 | 41 | 68.3±9.6 | 67.6±10.2 | 26 | 26 | 14 | 15 | 38 | 30 |
| Liao et al. | 38 | 36 | 66.9±9.0 | 67.2±8.6 | 22 | 18 | 18 | 16 | 30 | 28 |
| RANGER II SFA | 278 | 98 | 70.6±9.5 | 69.1±10.3 | 173 | 67 | 237 | 83 | 251 | 80 |
| BIOPAC | 33 | 33 | 65.2±9.4 | 65.9±7.8 | 30 | 23 | 15 | 10 | 27 | 30 |
| Ni et al. | 93 | 99 | 68.8±8.3 | 68.1±10.5 | 67 | 71 | 47 | 48 | 63 | 68 |
| ORCHID CHINA | 30 | 30 | 69.2±9.0 | 68.3±8.6 | 18 | 20 | 11 | 14 | 25 | 27 |
| Ye et al. | 100 | 100 | 67.8±9.2 | 69.4±10.3 | 72 | 73 | 31 | 27 | 73 | 75 |
| FREEWAY-CHINA | 155 | 154 | 69.87±9.63 | 70.7±8.57 | 116 | 102 | 73 | 71 | 108 | 121 |
| EMINENT | 508 | 167 | 68.9±8.7 | 68.9±9.1 | 363 | 180 | 384 | 207 | 397 | 203 |
|  | | | | | | | | | | |
|  | T,Hyperlipidemia | C,Hyperlipidemia | T,Diabetes | C,Diabetes | T,Renal insufficiency | C,Renal insufficiency | T, History of PAD | C, History of PAD | T,CAD | C,CAD |
| ZILVER-PTX | 180 | 166 | 117 | 100 | 24 | 25 | NA | NA | 50 | 41 |
| THUNDER | 33 | 34 | 24 | 25 | NA | NA | NA | NA | NA | NA |
| IN.PACT SFA | 186 | 91 | 89 | 54 | NA | NA | NA | NA | 122 | 60 |
| FEMPAC | 26 | 24 | 18 | 23 | NA | NA | NA | NA | NA | NA |
| LEVANT I | 29 | 36 | 22 | 26 | NA | NA | 32 | 28 | 19 | 23 |
| LEVANT II | 283 | 138 | 137 | 67 | 11 | 7 | NA | NA | 157 | 77 |
| ILLUMENATE EU | 137 | 49 | 83 | 26 | 20 | 6 | NA | NA | 29 | 12 |
| CONSEQUENT | 44 | 39 | 27 | 29 | 2 | 4 | NA | NA | 33 | 30 |
| ISAR-STATH | 45 | 45 | 10 | 15 | NA | NA | NA | NA | 25 | 24 |
| ISAR-PEBIS | 35 | 33 | 12 | 12 | NA | NA | NA | NA | 17 | 16 |
| IN.PACT SFA JAPAN | NA | NA | 40 | 18 | 6 | 4 | 39 | 19 | 34 | 16 |
| ACOART I | 27 | 29 | 54 | 57 | NA | NA | NA | NA | NA | NA |
| FINN-PTX | 13 | 15 | 9 | 6 | NA | NA | NA | NA | 6 | 5 |
| BATTLE | 55 | 61 | 41 | 22 | 8 | 6 | NA | NA | 27 | 34 |
| DEBATE-IN-SFA | 52 | 49 | 50 | 48 | 17 | 19 | NA | NA | 44 | 34 |
| DEBELLUM | 12 | 17 | 13 | 9 | NA | NA | NA | NA | NA | NA |
| PACIFIER | 22 | 22 | 19 | 13 | NA | NA | NA | NA | 14 | 15 |
| FAIR | 48 | 45 | 28 | 17 | 8 | 10 | 16 | 12 | 26 | 22 |
| BIOLUX P-I | 18 | 19 | 11 | 9 | NA | NA | 17 | 18 | 8 | 11 |
| RANGER SFA | 49 | 21 | 28 | 12 | 8 | 1 | NA | NA | 24 | 13 |
| ILLUMENATE pivotal | 176 | 90 | 99 | 52 | 36 | 16 | NA | NA | 90 | 48 |
| DEBATE-SFA | 33 | 27 | 41 | 36 | NA | NA | NA | NA | 21 | 18 |
| LEVANT JAPAN | 47 | 26 | 33 | 18 | 5 | 2 | NA | NA | 31 | 14 |
| RAPID | NA | NA | 23 | 24 | NA | NA | NA | NA | NA | NA |
| EFFPAC | 60 | 59 | 31 | 35 | NA | NA | NA | NA | NA | NA |
| PACUBA | 18 | 25 | 17 | 13 | 6 | 6 | NA | NA | NA | NA |
| FREEWAY | 63 | 57 | 28 | 26 | NA | NA | 39 | 44 | 26 | 23 |
| DRECOREST | 25 | 27 | 11 | 17 | 6 | 4 | NA | NA | 14 | 11 |
| SWEDEPAD | NA | NA | 519 | 508 | 253 | 260 | NA | NA | 521 | 487 |
| Falkowski et al. | 74 | 86 | 46 | 52 | 14 | 16 | NA | NA | 56 | 66 |
| COPA CABANA | 29 | 32 | 20 | 19 | NA | NA | NA | NA | 10 | 10 |
| Liao et al. | 25 | 24 | 19 | 17 | NA | NA | NA | NA | 13 | 13 |
| RANGER II SFA | 211 | 78 | 118 | 43 | 30 | 5 | NA | NA | 131 | 44 |
| BIOPAC | 20 | 17 | 11 | 11 | NA | NA | NA | NA | 19 | 19 |
| Ni et al. | 26 | 22 | 46 | 46 | NA | NA | NA | NA | NA | NA |
| ORCHID CHINA | 24 | 22 | 14 | 16 | NA | NA | NA | NA | 14 | 12 |
| Ye et al. | 20 | 13 | 58 | 47 | NA | NA | NA | NA | 71 | 79 |
| FREEWAY-CHINA | 20 | 24 | 101 | 77 | 2 | 1 | 55 | 64 | 49 | 43 |
| EMINENT | 341 | 182 | 162 | 87 | 59 | 22 | NA | NA | NA | NA |
|  | | | | | | | | | | |
|  | T,Carotid Artery Disease | C,Carotid Artery Disease | T,Cerebrovascular disease | C,Cerebrovascular disease | T,IC | C,IC | T,CLTI | C,CLTI | T,ABI | C,ABI |
| ZILVER-PTX | NA | NA | NA | NA | 217 | 216 | 24 | 22 | 0.67±0.2 | 0.68±0.2 |
| THUNDER | NA | NA | NA | NA | 35 | 47 | 13 | 7 | 0.5±0.3 | 0.5±0.3 |
| IN.PACT SFA | 73 | 32 | NA | NA | 209 | 104 | 11 | 7 | 0.769±0.228 | 0.744±0.189 |
| FEMPAC | NA | NA | NA | NA | 43 | 39 | 2 | 3 | 0.7/0.6–0.8 | 0.7/0.5–0.8 |
| LEVANT I | NA | NA | NA | NA | 46 | 48 | 3 | 4 | 0.69±0.23 | 0.60±0.36 |
| LEVANT II | NA | NA | NA | NA | 291 | 147 | 25 | 13 | 0.74±0.20 | 0.73±0.18 |
| ILLUMENATE EU | NA | NA | 38 | 15 | 217 | 70 | 4 | 1 | 0.72±0.21 | 0.69±0.26 |
| CONSEQUENT | NA | NA | NA | NA | 78 | 75 | 0 | 0 | 0.83 ± 0.17 | 0.82 ± 0.14 |
| ISAR-STATH | NA | NA | NA | NA | 45 | 48 | 3 | 4 | 0.7±0.3 | 0.8±0.4 |
| ISAR-PEBIS | NA | NA | NA | NA | 35 | 33 | 1 | 1 | 0.6±0.3 | 0.7±0.2 |
| IN.PACT SFA JAPAN | 12 | 5 | NA | NA | 65 | 31 | 3 | 1 | 0.76±0.15 | 0.74±0.17 |
| ACOART I | NA | NA | NA | NA | 60 | 56 | 40 | 44 | 0.49±0.24 | 0.43±0.28 |
| FINN-PTX | NA | NA | 3 | 2 | 17 | 17 | 6 | 1 | 0.54 (Range 0-0.82) | 0.65 (Range 0.47-0.99) |
| BATTLE | NA | NA | 11 | 9 | 68 | 70 | 18 | 15 | NA | NA |
| DEBATE-IN-SFA | NA | NA | 19 | 20 | 79 | 75 | 6 | 10 | 0.68 (IQR 0.58-0.77) | 0.65 (IQR 0.57-0.75) |
| DEBELLUM | NA | NA | NA | NA | 23 | 22 | 2 | 3 | 0.55±0.06 | 0.57±0.05 |
| PACIFIER | NA | NA | 14 | 5 | 42 | 45 | 2 | 2 | 0.73±0.30 | 0.65±0.26 |
| FAIR | 14 | 10 | NA | NA | 59 | 51 | 3 | 6 | 0.63±0.27 | 0.64±0.25 |
| BIOLUX P-I | NA | NA | 6 | 6 | 24 | 26 | 6 | 4 | 0.7±0.2 | 0.7±0.2 |
| RANGER SFA | NA | NA | NA | NA | 71 | 32 | 0 | 2 | 0.96±0.16 | 0.93±0.22 |
| ILLUMENATE pivotal | NA | NA | 47 | 20 | 192 | 95 | 8 | 5 | 0.75±0.21 | 0.76±0.20 |
| DEBATE-SFA | NA | NA | 11 | 9 | 11 | 16 | 42 | 35 | 0.33±0.22 | 0.31±0.18 |
| LEVANT JAPAN | NA | NA | 13 | 9 | 71 | 37 | 0 | 1 | 0.7±0.1 | 0.7±0.1 |
| RAPID | NA | NA | NA | NA | 66 | 67 | 14 | 13 | 0.59±0.20 | 0.61±0.19 |
| EFFPAC | NA | NA | 6 | 3 | 82 | 85 | 3 | 1 | 0.73±0.23 | 0.74±0.23 |
| PACUBA | NA | NA | NA | NA | 35 | 39 | 0 | 0 | 0.65±0.16 | 0.65±0.16 |
| FREEWAY | NA | NA | NA | NA | 98 | 96 | 7 | 3 | NA | NA |
| DRECOREST | NA | NA | 4 | 2 | 13 | 18 | 16 | 10 | 0.6 (Range 0-1) | 0.74 (Range 0-1.24) |
| SWEDEPAD | NA | NA | 161 | 152 | 404 | 405 | 745 | 735 | 0.6 (IQR 0.4–0.7) | 0.6 (IQR 0.4–0.7) |
| Falkowski et al. | NA | NA | 14 | 12 | NA | NA | NA | NA | NA | NA |
| COPA CABANA | NA | NA | 14 | 6 | 44 | 33 | 3 | 5 | 0.72±0.23 | 0.65±0.25 |
| Liao et al. | 9 | 7 | NA | NA | 21 | 23 | 17 | 13 | 0.50±0.13 | 0.52±0.13 |
| RANGER II SFA | NA | NA | NA | NA | 252 | 91 | 26 | 7 | 0.8±0.2 | 0.8±0.2 |
| BIOPAC | NA | NA | NA | NA | 28 | 29 | 5 | 5 | 0.5±0.2 | 0.6±0.2 |
| Ni et al. | NA | NA | NA | NA | 35 | 30 | 37 | 32 | 0.57±0.28 | 0.59±0.34 |
| ORCHID CHINA | 6 | 8 | NA | NA | 18 | 18 | 12 | 12 | 0.54±0.12 | 0.50±0.13 |
| Ye et al. | NA | NA | NA | NA | 74 | 72 | 26 | 28 | NA | NA |
| FREEWAY-CHINA | NA | NA | 39 | 39 | 112 | 43 | 103 | 51 | NA | NA |
| EMINENT | NA | NA | NA | NA | NA | NA | NA | NA | NA | NA |

Values are mean±standard error for continuous variables without additional descriptions.

ABI: ankle–brachial index; C: control; CAD: coronary artery disease; CLTI: chronic limb threatening ischemia; IC: intermittent claudication; IQR: interquartile range; NA: not available; T: treatment (paclitaxel).
