# Supplementary material for: Safety of paclitaxel-coated devices in the femoropopliteal arteries: A systematic review and meta-analysis
Source: PLoS One. 2022 Oct 13;17(10):e0275888. doi: 10.1371/journal.pone.0275888 (PMC9560511; doi:10.1371/journal.pone.0275888)
Supplement: S3 Table — (DOCX) [file pone.0275888.s005.docx]

**S3 Table. Detailed angiographic statistics of the 39 included randomized controlled trials.**

| TrialName | T,Lesions,n | C,Lesions,n | T,Lesions,Length (cm) | C,Lesions,Length (cm) | T,Vessel Diameter (mm) | C,Vessel Diameter (mm) | T, Occlusions | C, Occlusions |
| --- | --- | --- | --- | --- | --- | --- | --- | --- |
| ZILVER-PTX | 247 | 251 | 6.64±3.89 | 6.32±4.05 | NA | NA | 79 | 68 |
| THUNDER | 86 | 86 | 7.5±6.2 | 7.4±6.7 | 5.0±0.7 | 4.7±0.6 | 13 | 14 |
| IN.PACT SFA | 221 | 113 | 8.94±4.89 | 8.81±5.12 | 4.647±0.841 | 4.681±0.828 | 57 | 22 |
| FEMPAC | 44 | 42 | 4.0 (IQR 2.1–6.1) | 4.7 (IQR 2.7–8.5) | 5.2 (IQR 4.9–6.2) | 5.0 (IQR 4.7–5.6) | 6 | 8 |
| LEVANT I | 49 | 52 | 8.08±3.70 | 8.02±3.78 | 4.1±0.6 | 4.2±0.7 | 20 | 22 |
| LEVANT II | 322 | 165 | 6.28±4.10 | 6.28±4.10 | 4.8±0.8 | 4.8±0.8 | 65 | 35 |
| ILLUMENATE EU | 254 | 79 | 7.2±5.2 | 7.1±5.3 | 5.0±0.8 | 4.8±0.7 | 48 | 15 |
| CONSEQUENT | 87 | 84 | 13.7±12.2 | 12.6±8.2 | 5.06 ± 0.77 | 5.38 ± 0.94 | 18 | 22 |
| ISAR-STATH | 48 | 52 | 6.8±4.4 | 7.4±5.6 | 5.0±1.0 | 5.0±0.9 | 28 | 35 |
| ISAR-PEBIS | 36 | 34 | 13.2±6.5 | 14.6±6.9 | 5.0±1.1 | 4.7±0.9 | 13 | 10 |
| IN.PACT SFA JAPAN | 68 | 32 | 9.15±5.85 | 8.89±6.01 | 4.84±0.75 | 4.68±0.66 | 11 | 5 |
| ACOART I | 100 | 100 | 14.7±11.0 | 15.2±10.9 | 3.83±0.57 | 3.74±0.83 | 57 | 52 |
| FINN-PTX | 23 | 18 | 13.2 (Range 5.0-25.0) | 11.3 (Range 5.0-19.6) | NA | NA | 22 | 23 |
| BATTLE | 86 | 85 | 7.3±3.2 | 7.3±3.9 | 5.8±0.6 | 5.8±0.5 | 30 | 33 |
| DEBATE-IN-SFA | 85 | 85 | 11.05 (IQR 4.93-24.90) | 9.60 (IQR 4.49-18.40) | 5.3±1.0 | 5.1±1.1 | 43 | 31 |
| DEBELLUM | 44 | 48 | 7.6±0.6 | 7.8±0.7 | NA | NA | 5 | 9 |
| PACIFIER | 44 | 47 | 7.0±5.3 | 6.6±5.5 | 4.9±1.3 | 4.9±1.3 | 10 | 18 |
| FAIR | 62 | 57 | 8.2±7.1 | 8.1±6.6 | 5.1±0.9 | 5.4±0.5 | 15 | 19 |
| BIOLUX P-I | 33 | 35 | 5.14±4.72 | 6.85±5.70 | 4.6±0.8 | 4.7±0.9 | NA | NA |
| RANGER SFA | 70 | 32 | 6.8±4.6 | 6.0±4.8 | 5.0±0.9 | 4.5±0.8 | 24 | 11 |
| ILLUMENATE pivotal | 200 | 100 | 8.0±4.5 | 8.9±4.6 | 4.9±0.9 | 5.2±1.1 | 38 | 18 |
| DEBATE-SFA | 55 | 55 | 9.4±6.0 | 9.6±6.9 | 5.0±0.5 | 5.1±0.5 | 30 | 38 |
| LEVANT JAPAN | 72 | 40 | 6.77±4.35 | 5.53±5.10 | 4.9±0.7 | 4.7±0.7 | 13 | 2 |
| RAPID | 80 | 80 | 15.8±7.4 | 15.8±7.6 | 5.1±0.7 | 5.2±0.8 | 61 | 56 |
| EFFPAC | 85 | 86 | 5.91±4.34 | 5.58±3.91 | 5.4±0.6 | 5.4±0.7 | 17 | 22 |
| PACUBA | 35 | 39 | 17.3±11.3 | 18.4±8.8 | 5.7±1.0 | 5.4±0.9 | 11 | 11 |
| FREEWAY | 105 | 99 | 7.7±4.2 | 8.3±4.1 | 4.7 ± 0.8 | 4.6 ± 0.9 | 67 | 63 |
| DRECOREST | 29 | 28 | 11.5 (Range 2-40) | 14.4 (Range 2-100) | 4.2 (Range 2.5-6) | 5.0 (Range 3-5.5) | 0 | 0 |
| SWEDEPAD | 1259 | 1297 | NA | NA | NA | NA | NA | NA |
| Falkowski et al. | 126 | 130 | 9.38±2.60 | 12.76±4.97 | NA | NA | 4 | 9 |
| COPA CABANA | 49 | 42 | 15.2±8.5 | 12.8±8.4 | 5.2±0.6 | 5.1±0.8 | 12 | 15 |
| Liao et al. | 56 | 56 | 17.9±8.0 | 18.2±9.1 | 5.9±0.7 | 6.0±0.9 | 16 | 15 |
| RANGER II SFA | 278 | 98 | 8.25±4.89 | 7.99±4.93 | 5.1±0.9 | 5.1±0.9 | 1 | 0 |
| BIOPAC | 34 | 35 | 6.50±4.53 | 5.85±4.97 | NA | NA | 15 | 9 |
| Ni et al. | 93 | 99 | 7.03±6.33 | 5.46±5.41 | 4.9±0.9 | 4.8±0.9 | 27 | 44 |
| ORCHID CHINA | 30 | 30 | 9.12±8.09 | 9.26±8.03 | 4.98±0.89 | 4.76±0.96 | 19 | 20 |
| Ye et al. | 100 | 100 | 9.6±4.8 | 9.1±4.4 | 3.9±0.8 | 3.9±0.8 | 49 | 55 |
| FREEWAY-CHINA | 183 | 166 | 6.99±4.03 | 7.22±4.03 | 4.87±0.61 | 4.89±0.59 | NA | NA |
| EMINENT | 508 | 267 | 7.56±5.03 | 7.22±4.70 | NA | NA | 215 | 107 |
|  | | | | | | | | |
|  | T,Calcification | C,Calcification | T, Lesion Location, SFA | C, Lesion Location, SFA | T, Lesion Location, Politeal | T, Lesion Location, Politeal | T, Lesion Location, SFA/Politeal | C, Lesion Location, SFA/Politeal |
| ZILVER-PTX | 226 | 232 | 232 | 229 | 13 | 9 | 6 | 9 |
| THUNDER | 24 | 28 | 33 | 35 | 15 | 19 | NA | NA |
| IN.PACT SFA | 18 | 7 | NA | NA | NA | NA | NA | NA |
| FEMPAC | 24 | 22 | NA | NA | NA | NA | NA | NA |
| LEVANT I | NA | NA | 45 | 49 | 4 | 3 | NA | NA |
| LEVANT II | 187 | 93 | 285 | 148 | 31 | 12 | NA | NA |
| ILLUMENATE EU | 111 | 32 | 224 | 69 | 27 | 10 | NA | NA |
| CONSEQUENT | NA | NA | 63 | 59 | 4 | 5 | 11 | 11 |
| ISAR-STATH | 43 | 44 | 48 | 52 | 0 | 0 | NA | NA |
| ISAR-PEBIS | NA | NA | 36 | 34 | 0 | 0 | NA | NA |
| IN.PACT SFA JAPAN | 5 | 3 | 67 | 31 | 1 | 1 | NA | NA |
| ACOART I | NA | NA | 74 | 76 | 11 | 13 | 15 | 11 |
| FINN-PTX | NA | NA | 23 | 18 | 0 | 0 | NA | NA |
| BATTLE | NA | NA | 106 | 87 | 11 | 15 | NA | NA |
| DEBATE-IN-SFA | 49 | 49 | 70 | 70 | 15 | 15 | NA | NA |
| DEBELLUM | NA | NA | 42 | 46 | 2 | 2 | NA | NA |
| PACIFIER | 28 | 31 | NA | NA | NA | NA | NA | NA |
| FAIR | 30 | 19 | 78 | 68 | 7 | 5 | NA | NA |
| BIOLUX P-I | NA | NA | 28 | 27 | 2 | 6 | NA | NA |
| RANGER SFA | 61 | 27 | 68 | 31 | 2 | 1 | NA | NA |
| ILLUMENATE pivotal | 87 | 43 | 191 | 91 | 9 | 9 | NA | NA |
| DEBATE-SFA | 22 | 19 | 41 | 45 | 14 | 10 | NA | NA |
| LEVANT JAPAN | 33 | 23 | NA | NA | NA | NA | NA | NA |
| RAPID | NA | NA | 80 | 80 | 0 | 0 | NA | NA |
| EFFPAC | 38 | 48 | 75 | 74 | 28 | 26 | NA | NA |
| PACUBA | NA | NA | NA | NA | NA | NA | NA | NA |
| FREEWAY | NA | NA | 103 | 99 | 2 | 0 | NA | NA |
| DRECOREST | NA | NA | NA | NA | NA | NA | NA | NA |
| SWEDEPAD | NA | NA | NA | NA | NA | NA | NA | NA |
| Falkowski et al. | NA | NA | NA | NA | NA | NA | NA | NA |
| COPA CABANA | 14 | 10 | 33 | 26 | 14 | 15 | NA | NA |
| Liao et al. | 16 | 19 | 48 | 50 | 8 | 6 | NA | NA |
| RANGER II SFA | NA | NA | 263 | 94 | 15 | 4 | NA | NA |
| BIOPAC | NA | NA | 34 | 33 | 7 | 4 | NA | NA |
| Ni et al. | 43 | 40 | 81 | 77 | 9 | 18 | 3 | 6 |
| ORCHID CHINA | 16 | 14 | NA | NA | NA | NA | NA | NA |
| Ye et al. | NA | NA | 83 | 87 | 17 | 13 | NA | NA |
| FREEWAY-CHINA | NA | NA | 168 | 147 | 15 | 19 | NA | NA |
| EMINENT | 442 | 234 | NA | NA | NA | NA | NA | NA |
|  | | | | | | | | |
|  | T, Lesion Type, De novo | C, Lesion Type, De novo | T, Lesion Type, Restenotic | C, Lesion Type, Restenotic | T,Dissection | C,Dissection | T, Bailout stenting | C, Bailout stenting |
| ZILVER-PTX | NA | NA | NA | NA | NA | NA | NA | NA |
| THUNDER | NA | NA | NA | NA | NA | NA | NA | NA |
| IN.PACT SFA | 209 | 105 | 11 | 6 | 80 | 44 | 16 | 14 |
| FEMPAC | 29 | 28 | 16 | 14 | NA | NA | NA | NA |
| LEVANT I | 44 | 46 | 5 | 6 | 9 | 10 | 1 | 6 |
| LEVANT II | 265 | 140 | 51 | 20 | 199 | 114 | 8 | 11 |
| ILLUMENATE EU | 234 | 71 | 20 | 8 | 1 | 0 | 39 | 9 |
| CONSEQUENT | NA | NA | NA | NA | NA | NA | NA | NA |
| ISAR-STATH | 48 | 52 | 0 | 0 | NA | NA | 48 | 52 |
| ISAR-PEBIS | 36 | 34 | 0 | 0 | NA | NA | 2 | 6 |
| IN.PACT SFA JAPAN | 62 | 32 | 6 | 0 | NA | NA | 3 | 1 |
| ACOART I | NA | NA | NA | NA | NA | NA | 19 | 21 |
| FINN-PTX | NA | NA | NA | NA | NA | NA | NA | NA |
| BATTLE | 86 | 85 | 0 | 0 | NA | NA | NA | NA |
| DEBATE-IN-SFA | 85 | 85 | 0 | 0 | NA | NA | 20 | 19 |
| DEBELLUM | NA | NA | NA | NA | NA | NA | NA | NA |
| PACIFIER | NA | NA | NA | NA | 18 | 25 | NA | NA |
| FAIR | 62 | 57 | 0 | 0 | NA | NA | 1 | 4 |
| BIOLUX P-I | NA | NA | NA | NA | 19 | 17 | 2 | 8 |
| RANGER SFA | NA | NA | NA | NA | NA | NA | NA | NA |
| ILLUMENATE pivotal | 181 | 82 | 19 | 18 | 0 | 0 | 12 | 6 |
| DEBATE-SFA | NA | NA | NA | NA | NA | NA | NA | NA |
| LEVANT JAPAN | 68 | 36 | 3 | 2 | NA | NA | NA | NA |
| RAPID | 80 | 80 | 0 | 0 | NA | NA | NA | NA |
| EFFPAC | NA | NA | NA | NA | 32 | 35 | 15 | 19 |
| PACUBA | NA | NA | NA | NA | NA | NA | NA | NA |
| FREEWAY | NA | NA | NA | NA | NA | NA | NA | NA |
| DRECOREST | NA | NA | NA | NA | NA | NA | NA | NA |
| SWEDEPAD | NA | NA | NA | NA | NA | NA | NA | NA |
| Falkowski et al. | NA | NA | NA | NA | NA | NA | NA | NA |
| COPA CABANA | NA | NA | NA | NA | NA | NA | NA | NA |
| Liao et al. | NA | NA | NA | NA | NA | NA | NA | NA |
| RANGER II SFA | NA | NA | NA | NA | 208 | 58 | NA | NA |
| BIOPAC | 31 | 2 | 30 | 3 | 8 | 7 | 13 | 13 |
| Ni et al. | 80 | 88 | 13 | 11 | NA | NA | 6 | 17 |
| ORCHID CHINA | NA | NA | NA | NA | 20 | 19 | 1 | 2 |
| Ye et al. | NA | NA | NA | NA | NA | NA | 21 | 22 |
| FREEWAY-CHINA | 161 | 148 | 22 | 18 | 36 | 29 | 40 | 47 |
| EMINENT | NA | NA | NA | NA | NA | NA | NA | NA |

Values are mean±standard error for continuous variables without additional descriptions.

C: control; IQR: interquartile range; NA: not available; T: treatment (paclitaxel).
