# Supplementary material for: Safety of paclitaxel-coated devices in the femoropopliteal arteries: A systematic review and meta-analysis
Source: PLoS One. 2022 Oct 13;17(10):e0275888. doi: 10.1371/journal.pone.0275888 (PMC9560511; doi:10.1371/journal.pone.0275888)
Supplement: S4 Table — (DOCX) [file pone.0275888.s006.docx]

**S4 Table. All-cause mortality at one, two and five years of the 39 included randomized controlled trials.**

| **Trial Name** | **Period**  **(Year)** | **Event (Paclitaxel)** | **Total (Paclitaxel)** | **Event**  **(Control)** | **Total**  **(Control)** | **Publication Year** |
| --- | --- | --- | --- | --- | --- | --- |
| ACOART I | 1 | 2 | 100 | 2 | 100 | 2016 |
| ACOART I | 2 | 8 | 96 | 6 | 95 | 2018 |
| ACOART I | 5 | 17 | 89 | 24 | 91 | 2021 |
| BATTLE | 1 | 1 | 86 | 2 | 85 | 2018 |
| BATTLE | 2 | 1 | 77 | 7 | 75 | 2020 |
| BIOLUX P-I | 1 | 0 | 30 | 2 | 30 | 2015 |
| CONSEQUENT | 1 | 2 | 70 | 1 | 65 | 2017 |
| CONSEQUENT | 2 | 2 | 70 | 1 | 65 | 2018 |
| COPA CABANA | 1 | 2 | 47 | 1 | 41 | 2020 |
| COPA CABANA | 2 | 2 | 47 | 3 | 41 | 2020 |
| DEBATE-IN-SFA | 1 | 5 | 84 | 1 | 85 | 2018 |
| DEBATE-SFA | 1 | 2 | 53 | 1 | 51 | 2013 |
| DEBELLUM | 1 | 0 | 25 | 0 | 25 | 2014 |
| DRECOREST | 1 | 4 | 29 | 2 | 28 | 2019 |
| EFFPAC | 1 | 1 | 85 | 2 | 86 | 2018 |
| EFFPAC | 2 | 1 | 85 | 2 | 86 | 2020 |
| EFFPAC | 5 | 9 | 80 | 14 | 86 | 2022 |
| FAIR | 1 | 2 | 47 | 3 | 44 | 2015 |
| Falkowski et al. | 1 | 1 | 126 | 1 | 130 | 2020 |
| Falkowski et al. | 2 | 6 | 126 | 4 | 130 | 2020 |
| FEMPAC | 1 | 1 | 45 | 0 | 42 | 2008 |
| FEMPAC | 2 | 7 | 45 | 3 | 42 | 2008 |
| FINN-PTX | 1 | 0 | 23 | 0 | 18 | 2018 |
| FINN-PTX | 2 | 1 | 23 | 0 | 18 | 2018 |
| FREEWAY | 1 | 1 | 90 | 2 | 81 | 2019 |
| ILLUMENATE EU | 1 | 6 | 215 | 1 | 66 | 2017 |
| ILLUMENATE EU | 2 | 16 | 214 | 3 | 65 | 2018 |
| ILLUMENATE EU | 5 | 40 | 207 | 13 | 67 | 2021 |
| ILLUMENATE pivotal | 1 | 5 | 200 | 2 | 100 | 2017 |
| ILLUMENATE pivotal | 2 | 13 | 200 | 8 | 99 | 2021 |
| ILLUMENATE pivotal | 5 | 39 | 184 | 18 | 89 | 2021 |
| IN.PACT SFA | 1 | 4 | 207 | 0 | 107 | 2014 |
| IN.PACT SFA | 2 | 16 | 198 | 1 | 106 | 2015 |
| IN.PACT SFA | 5 | 29 | 184 | 10 | 104 | 2019 |
| IN.PACT SFA JAPAN | 1 | 0 | 68 | 0 | 32 | 2018 |
| IN.PACT SFA JAPAN | 2 | 4 | 66 | 1 | 29 | 2019 |
| ISAR-PEBIS | 1 | 1 | 33 | 0 | 33 | 2017 |
| ISAR-PEBIS | 2 | 3 | 28 | 0 | 29 | 2017 |
| ISAR-STATH | 1 | 1 | 48 | 0 | 107 | 2017 |
| ISAR-STATH | 2 | 3 | 48 | 1 | 107 | 2017 |
| LEVANT I | 1 | 2 | 49 | 4 | 52 | 2014 |
| LEVANT I | 2 | 4 | 49 | 5 | 52 | 2014 |
| LEVANT II | 1 | 7 | 290 | 4 | 144 | 2015 |
| LEVANT II | 2 | 19 | 285 | 8 | 146 | 2018 |
| LEVANT II | 5 | 54 | 266 | 17 | 137 | 2019 |
| Liao et al. | 1 | 1 | 38 | 2 | 36 | 2019 |
| LEVANT JAPAN | 1 | 1 | 71 | 1 | 38 | 2019 |
| LEVANT JAPAN | 2 | 2 | 71 | 3 | 38 | 2019 |
| PACIFIER | 1 | 0 | 42 | 3 | 43 | 2012 |
| PACIFIER | 2 | 2 | 39 | 4 | 42 | 2019 |
| PACUBA | 1 | 0 | 35 | 0 | 39 | 2016 |
| RANGER II SFA | 1 | 5 | 259 | 2 | 93 | 2021 |
| RANGER II SFA | 2 | 15 | 261 | 3 | 93 | 2022 |
| RANGER SFA | 1 | 2 | 71 | 1 | 34 | 2018 |
| RANGER SFA | 2 | 6 | 71 | 3 | 34 | 2019 |
| RAPID | 1 | 1 | 80 | 2 | 80 | 2017 |
| RAPID | 2 | 4 | 80 | 4 | 80 | 2019 |
| SWEDEPAD | 1 | 117 | 1149 | 113 | 1140 | 2020 |
| SWEDEPAD | 2 | 215 | 1149 | 198 | 1140 | 2020 |
| THUNDER | 1 | 2 | 48 | 1 | 54 | 2008 |
| THUNDER | 2 | 5 | 48 | 7 | 54 | 2015 |
| THUNDER | 5 | 12 | 48 | 8 | 54 | 2015 |
| ZILVER-PTX | 1 | 9 | 297 | 4 | 177 | 2011 |
| ZILVER-PTX | 2 | 19 | 297 | 7 | 177 | 2013 |
| ZILVER-PTX | 5 | 47 | 302 | 18 | 177 | 2016 |
| BIOPAC | 1 | 0 | 33 | 3 | 33 | 2021 |
| BIOPAC | 2 | 0 | 33 | 3 | 33 | 2021 |
| Ni et al. | 1 | 3 | 93 | 2 | 99 | 2022 |
| ORCHID CHINA | 1 | 0 | 30 | 0 | 30 | 2021 |
| Ye et al. | 1 | 3 | 100 | 5 | 100 | 2021 |
| Ye et al. | 2 | 8 | 82 | 14 | 79 | 2021 |
| FREEWAY-CHINA | 1 | 7 | 155 | 4 | 154 | 2021 |
| EMINENT | 1 | 13 | 474 | 3 | 263 | 2022 |
