# Supplementary material for: Safety of paclitaxel-coated devices in the femoropopliteal arteries: A systematic review and meta-analysis
Source: PLoS One. 2022 Oct 13;17(10):e0275888. doi: 10.1371/journal.pone.0275888 (PMC9560511; doi:10.1371/journal.pone.0275888)
Supplement: S5 Table — Risk ratio (95% CI) for frequentist methods and odds ratio (95% equal-tailed CrI) for Bayesian methods. (DOCX) [file pone.0275888.s007.docx]

**S5 Table. Sensitivity analyses of all-cause mortality. Risk ratio (95% CI) for frequentist methods and odds ratio (95% equal-tailed CrI) for Bayesian methods.**

| **Period** | **Case** | **Random effects** | **Fixed effects** |
| --- | --- | --- | --- |
| 1-year | Continuity Correction 0.5 (Primary analysis) | 1.06 (0.87, 1.29) | 1.07 (0.88, 1.29) |
|  | Continuity Correction 0.01 | 1.06 (0.87, 1.30) | 1.07 (0.88, 1.30) |
|  | Mantel-Haenszel exact method | 1.06 (0.87, 1.29) | 1.07 (0.88, 1.30) |
|  | Treatment arm continuity correction | 1.06 (0.87, 1.29) | 1.07 (0.88, 1.29) |
|  | Bayesian binomial-logit model on arm-level observations | 1.08 (0.79, 1.47) | 1.06 (0.86, 1.31) |
|  | Bayesian binomial-logit model on arm-level observations (adjusted for the proportion of CLTI patients at baseline) | 1.06 (0.77, 1.47) | 1.05 (0.85, 1.31) |
|  | | | |
| 2-year | Continuity Correction 0.5 (Primary analysis) | 1.08 (0.93, 1.25) | 1.10 (0.95, 1.26) |
|  | Continuity Correction 0.5 (SWEDEPAD not included) | 1.08 (0.83, 1.42) | 1.13 (0.88, 1.44) |
|  | Continuity Correction 0.01 | 1.08 (0.93, 1.25) | 1.10 (0.95, 1.26) |
|  | Mantel-Haenszel exact method | 1.08 (0.93, 1.25) | 1.10 (0.95, 1.26) |
|  | Treatment arm continuity correction | 1.08 (0.93, 1.25) | 1.10 (0.95, 1.26) |
|  | Bayesian binomial-logit model on arm-level observations | 1.12 (0.83, 1.54) | 1.11 (0.94, 1.31) |
|  | Bayesian binomial-logit model on arm-level observations | 1.12 (0.81, 1.55) | 1.11 (0.94, 1.31) |
|  | | | |
| 5-year | Continuity Correction 0.5 (Primary analysis) | 1.18 (0.92, 1.51) | 1.20 (0.98, 1.48) |
|  | Continuity Correction 0.01 | 1.18 (0.92, 1.51) | 1.20 (0.98, 1.48) |
|  | Mantel-Haenszel exact method | 1.18 (0.92, 1.51) | 1.20 (0.98, 1.48) |
|  | Treatment arm continuity correction | 1.18 (0.92, 1.51) | 1.20 (0.98, 1.48) |
|  | Bayesian binomial-logit model on arm-level observations | 1.22 (0.81, 1.84) | 1.25 (0.98, 1.60) |
|  | Bayesian binomial-logit model on arm-level observations (adjusted for the proportion of CLTI patients at baseline) | 1.22 (0.81, 1.84) | 1.24 (0.98, 1.59) |

CI: confidence interval; CLTI: chronic limb threatening ischemia; CrI: credible interval.
