# Supplementary material for: Safety of paclitaxel-coated devices in the femoropopliteal arteries: A systematic review and meta-analysis
Source: PLoS One. 2022 Oct 13;17(10):e0275888. doi: 10.1371/journal.pone.0275888 (PMC9560511; doi:10.1371/journal.pone.0275888)
Supplement: S6 Table — (DOCX) [file pone.0275888.s008.docx]

**S6 Table. Cumulative meta-analysis by years of publications.**

| **Period (year)** | **Publication Year** | **No. of Study** | **RR (95% CI)** |
| --- | --- | --- | --- |
| 1 | 2011 | 3 | 1.58 (0.59 ,4.25) |
| 1 | 2012 | 4 | 1.24 (0.48 ,3.16) |
| 1 | 2013 | 5 | 1.31 (0.55 ,3.14) |
| 1 | 2014 | 8 | 1.19 (0.56 ,2.50) |
| 1 | 2015 | 11 | 0.96 (0.53 ,1.72) |
| 1 | 2016 | 13 | 0.96 (0.55 ,1.68) |
| 1 | 2017 | 19 | 1.09 (0.68 ,1.76) |
| 1 | 2018 | 25 | 1.10 (0.71 ,1.71) |
| 1 | 2019 | 29 | 1.07 (0.71 ,1.61) |
| 1 | 2020 | 32 | 1.04 (0.85 ,1.28) |
| 1 | 2021 | 37 | 1.03 (0.84 ,1.26) |
| 1 | 2022 | 39 | 1.06 (0.87 ,1.29) |
| 2 | 2014 | 3 | 1.48 (0.80 ,2.75) |
| 2 | 2015 | 5 | 1.49 (0.79 ,2.82) |
| 2 | 2016 | 5 | 1.49 (0.79 ,2.82) |
| 2 | 2017 | 7 | 1.78 (0.95 ,3.34) |
| 2 | 2018 | 12 | 1.51 (1.05 ,2.17) |
| 2 | 2019 | 17 | 1.32 (0.95 ,1.82) |
| 2 | 2020 | 22 | 1.11 (0.95 ,1.29) |
| 2 | 2021 | 25 | 1.07 (0.91 ,1.26) |
| 2 | 2022 | 26 | 1.08 (0.93 ,1.25) |
| 5 | 2019 | 4 | 1.61 (1.20 ,2.16) |
| 5 | 2020 | 4 | 1.61 (1.20 ,2.16) |
| 5 | 2021 | 7 | 1.23 (0.97 ,1.58) |
| 5 | 2022 | 8 | 1.18 (0.92 ,1.51) |

CI: confidence interval; RR: risk ratio.
