# Supplementary material for: Safety of paclitaxel-coated devices in the femoropopliteal arteries: A systematic review and meta-analysis
Source: PLoS One. 2022 Oct 13;17(10):e0275888. doi: 10.1371/journal.pone.0275888 (PMC9560511; doi:10.1371/journal.pone.0275888)
Supplement: S7 Table — (DOCX) [file pone.0275888.s009.docx]

**S7 Table. Egger’s test of publication bias**

| **Period** | **No. of studies** | **P value** |
| --- | --- | --- |
| 1 year | 34 | 0.865 |
| 2 years | 26 | 0.824 |
| 5 years | 8 | 0.886 |
