# Supplementary material for: Safety of paclitaxel-coated devices in the femoropopliteal arteries: A systematic review and meta-analysis
Source: PLoS One. 2022 Oct 13;17(10):e0275888. doi: 10.1371/journal.pone.0275888 (PMC9560511; doi:10.1371/journal.pone.0275888)
Supplement: Attachment — Submitted filename: Response to Reviewers.docx [file pone.0275888.s011.docx]

We thank you for your careful review of our manuscript and many insightful comments. We have revised our manuscript by considering all of your comments, which led to a much better exposition of our work. Our point-by-point responses to major comments are given below, with your original comments copied in Italics for your convenience.

**Comments:**

**To Editor:**

1. *In particular, carefully revise the tone of the discussion to accommodate the recommendations by Reviewer #3.*

**Response**: Thanks for your suggestion. We have revised the mentioned sentences in the Abstract and Discussion sections following the recommendations by Reviewer #3.

**To Reviewer #3**

1. *I suggest to re-consider the sentences were a trend towards increased risk of death is highlighted, since this is not strongly supported by the findings, especially when looking at more recently published RCTs (after 2020).*

**Response**: Thank you for pointing out this problem. In the revised manuscript we have modified sentences involving ‘a trend towards increased risk of death’ in the Abstract and Discussion sections.
